# Development of New Meridianin/Leucettine-Derived Hybrid Small Molecules as Nanomolar Multi-Kinase Inhibitors with Antitumor Activity

**DOI:** 10.3390/biomedicines9091131

**Published:** 2021-09-01

**Authors:** Mohamed H. Elsherbeny, Ahmed Elkamhawy, Hossam Nada, Magda H. Abdellattif, Kyeong Lee, Eun Joo Roh

**Affiliations:** 1 Chemical Kinomics Research Center, Korea Institute of Science and Technology (KIST), Seoul 02792, Korea; mohamed.alsherbeny@pharma.asu.edu.eg; 2 Division of Bio-Medical Science & Technology, KIST School, University of Science and Technology, Seoul 02792, Korea; 3 Pharmaceutical Chemistry Department, Faculty of Pharmacy, Ahram Canadian University, Giza 12566, Egypt; 4 College of Pharmacy, Dongguk University-Seoul, Goyang 10326, Korea; hossam_hammouda@dgu.ac.kr (H.N.); kaylee@dongguk.edu (K.L.); 5 Department of Pharmaceutical Organic Chemistry, Faculty of Pharmacy, Mansoura University, Mansoura 35516, Egypt; 6 Pharmaceutical Chemistry Department, Faculty of Pharmacy, Badr University, Cairo 11829, Egypt; 7 Department of Chemistry, College of Science, Taif University, P.O. Box 11099, Taif 21944, Saudi Arabia; m.hasan@tu.edu.sa

**Keywords:** meridianins, leucettine, marine-inspired kinase inhibitors, DAPK1, FMS, LCK, LYN, molecular modeling, ADME studies

## Abstract

Although the sea ecosystem offers a broad range of bioactivities including anticancer, none of the FDA-approved antiproliferative protein kinase inhibitors are derived from a marine source. In a step to develop new marine-inspired potent kinase inhibitors with antiproliferative activities, a new series of hybrid small molecules (**5a–5g**) was designed and synthesized based on chemical moieties derived from two marine natural products (Meridianin E and Leucettamine B). Over a panel of 14 cancer-related kinases, a single dose of 10 µM of the parent hybrid **5a** possessing the benzo[*d*][1,3]dioxole moiety of Leucettamine B was able to inhibit the activity of FMS, LCK, LYN, and DAPK1 kinases with 82.5 ± 0.6, 81.4 ± 0.6, 75.2 ± 0.0, and 55 ± 1.1%, respectively. Further optimization revealed the most potent multiple kinase inhibitor of this new series (**5g**) with IC_50_ values of 110, 87.7, and 169 nM against FMS, LCK, and LYN kinases, respectively. Compared to imatinib (FDA-approved multiple kinase inhibitor), compound **5g** was found to be ~ 9- and 2-fold more potent than imatinib over both FMS and LCK kinases, respectively. In silico docking simulation models of the synthesized compounds within the active site of FMS, LCK, LYN, and DAPK1 kinases offered reasonable explanations of the elicited biological activities. In an in vitro anticancer assay using a library of 60 cancer cell lines that include blood, lung, colon, CNS, skin, ovarian, renal, prostate, and breast cancers, it was found that compound **5g** was able to suppress 60 and 70% of tumor growth in leukemia SR and renal RXF 393 cell lines, respectively. Moreover, an ADME study indicated a suitable profile of compound **5g** concerning cell permeability and blood-brain barrier (BBB) impermeability, avoiding possible CNS side effects. Accordingly, compound **5g** is reported as a potential lead towards novel antiproliferative marine-derived kinase modulators.

## 1. Introduction

The process of drug development from marine organisms is a prehistoric praxis. To date, more than 20,000 marine natural products (MNPs) have been isolated from ocean life-forms. The discovery of novel small molecules based on a natural heterocyclic scaffold has always attracted the attention of medicinal chemists worldwide. This fact was driven by the broad range of bioactivities that the sea ecosystem offers such as anticancer, anti-inflammatory, antibacterial, antiviral, antifungal, antifouling, antiprotozoal, anticoagulant, immunosuppressive, and neuroprotective activities [1,2,3,4]. However, to date, only eight anticancer drugs of marine origin were approved by the US Food and Drug Administration (FDA), the European Medicines Evaluation Agency (EMEA), or the Australian Therapeutic Goods Administration (TGA), as well as a few in phases I, II, and III clinical pipelines [5,6].

Over the past two decades, drug development has shifted from the random screening of large compound libraries of synthetic origin using high-throughput cell-based cytotoxicity assays to screening against clinically validated molecular targets [7,8,9]. This new target-based discovery aims to enhance the efficacy and selectivity of treatment by offering new drug candidates that block disease mechanisms in a defined and specific way. This new approach is widely driven by the rapidly expanding knowledge of disease biology and pathology at the molecular level. This approach has been particularly successful in oncology [10,11]. Among these targets, protein kinases are involved in various cellular functions including metabolism, cell cycle regulation, survival, and differentiation.

Dysregulation of protein kinases is implicated in various processes of carcinogenesis [12]. Moreover, overexpression of various types of protein kinases is found in different types of cancer, which encouraged medicinal chemists worldwide to develop numerous receptor tyrosine kinases inhibitors (RTKIs). In addition, the advent of protein kinase inhibitors in cancer research and therapy has led to a paradigm shift in how cancer is currently treated [13,14,15,16,17,18,19,20,21,22,23,24,25,26,27,28,29]. As a result, the FDA has approved many protein kinase inhibitors in the last few decades. Surprisingly, none of them are derived from a marine source [1,30].

**Figure 1:**
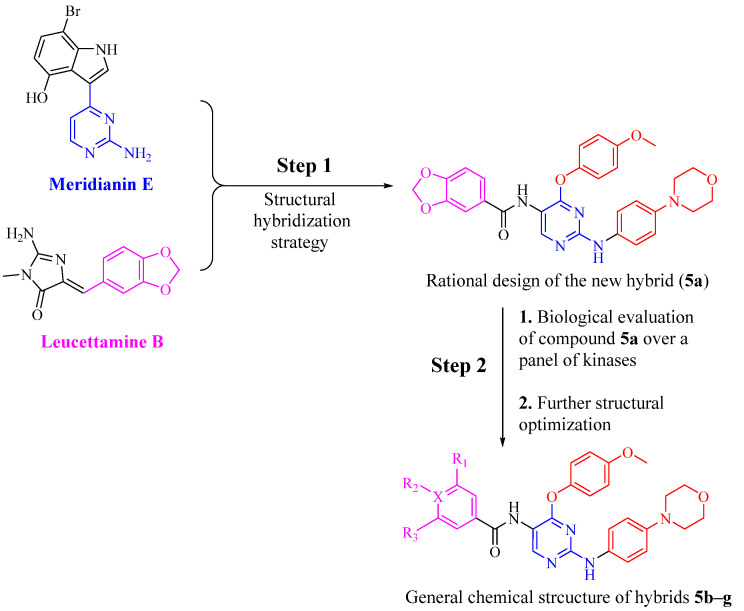
Rational design steps of the new set of Meridianin E and Leucettamine B hybrids (**5a–5g**).

Searching the literature reveals interesting kinase inhibitory activities of two MNPs (Meridianin E and Leucettamine B). Meridianins are indole alkaloids, isolated from tunicate *Aplidium meridianum*, inhibit various protein kinases associated with neurodegenerative and cancer diseases. These compounds also showed promising antiproliferative activity in several cancer cell lines. Amongst natural meridianins, meridianin E (Figure 1) attracted our attention since it exhibited significant cytotoxicity against murine tumor cell lines [31]. Moreover, it demonstrated potent and selective inhibition of CDK-1 and CDK-5 kinases. Furthermore, several synthetic meridianin analogs showed potent and selective inhibitory effects over glycogen synthase-3 (GSK-3) and dual-specificity tyrosine-phosphorylation regulated kinase 1A (DYRK-1A), which are known to be implicated in the progression of Alzheimer’s disease [2,32]. On the other hand, Leucettamine B (Figure 1) is a natural product found in marine sponge *Leucetta microraphis*. Several analogs of its family such as aplysinopsine and clathridine are medicinally active molecules that have applications in many pharmaceuticals and healthcare products. A recent study also reported the potential anticancer activity of a series of Leucettamine B synthesized derivatives [33]. However, leucettamine B and its analog leucettine L41 have not been well studied for their kinase inhibitory activity. Only a few reports in the literature indicated the ability of leucettamine B to inhibit “dual-specificity” kinases DYRK-1A, DYRK-2, CLK-1, and CLK-3 with high IC_50_ values of 2.8, 1.5, 0.40, and 6.4 μM, respectively [34,35,36,37]. Accordingly, with the great potential of these two MNPs (Meridianin E and Leucettamine B) to afford new more potent kinase inhibitors, further investigations in this field are highly needed. Thus, this encouraged us to apply a structure-based drug design strategy towards the development of a new marine-inspired potential kinase inhibitor (**5a**, Figure 1).

As shown in Figure 1, a structural hybridization approach was carried out by incorporating the pyrimidine scaffold of Meridianin E with the benzo[*d*][1,3]dioxole moiety of Leucettamine B via a backbone amide linker. The pyrimidine nucleus was also substituted at positions 2 and 4 with 4-morpholinophenylamino and 4-methoxyphenoxy moieties, respectively. These two substituents, widely found as solvent exposure moieties in the chemical structures of many kinase inhibitors, were introduced to enhance the binding interaction of the synthesized hybrid inhibitor with the binding site of the potential kinase target(s). The performed hybridization strategy led to the design and synthesis of the new hybrid small molecule **5a** which was assessed for its biological activity over a panel of 14 cancer-related kinases in a step to identify a potential kinase inhibitory activity. Optimization of the chemical structure of compound **5a** afforded new derivatives **5b–5g** which were further biologically evaluated for their kinase inhibitory and antiproliferative activities. Compounds that showed inhibition > 50% over any tested kinase were further assessed for their IC_50_ on the corresponding kinase. Moreover, the target compounds were tested for their in vitro cytotoxic activity against the NCI 60 cell lines panel. Molecular docking studies were also carried out for the designed compounds with the target kinases to study their binding modes and their interactions with the key amino acids in the ATP-binding pocket. Accordingly, we report our rational design, optimization, synthetic routes, in vitro and in silico biological evaluation of the newly synthesized marine-derived compounds **5a–5g**.

## 2. Materials and Methods

### 2.1. Chemistry

#### General

All reagents and solvents were purchased from TCI, Sigma-Aldrich, and Alfa Aesar, and were used without further purification. Biotage Initiator+ apparatus was used to carry out microwave-assisted reactions (Biotage AB, Uppsala, Sweden). Sealed vessels with magnetic stirrers were used to perform the reactions under controlled temperature for a programmed duration. The chemical synthesis, column chromatography, NMR identification, purity, and HRMS experiments were carried out following the previously reported general methods [38,39] (for details, see Supplementary File).

***Synthesis of 2-chloro-4-(4-methoxyphenoxy)-5-nitropyrimidine (2)***. A solution of 4-methoxyphenol (10 mmol) dissolved in a mixture of 1N aqueous sodium bicarbonate (10 mL) and water (40 mL) was added dropwise using an addition funnel to a 250 mL rounded-bottom flask containing a prepared solution of 2,4-dichloro-5-nitro-pyrimidine (10 mmol) dissolved in acetone (50 mL) and cooled to 0 °C. The flask was then allowed to return to room temperature and kept under stirring for 3 h until TLC showed the reaction completion. The reaction mixture was evaporated under vacuum, and the residue was washed sequentially with EA, 1N NaOH (aq.), and brine. The organic layer was then dried over anhydrous Na_2_SO_4_ and purified using flash chromatography (20% EA/Hex) to obtain compound **2**. Yellowish white solid, yield: 87%, ^1^H NMR (400 MHz, DMSO-*d*_6_): δ 9.39 (s, 1H), 7.24 (d, *J* = 9.2 Hz, 2H), 7.05 (d, *J* = 9.2 Hz, 2H), 3.81 (s, 3H). Reported [18].

***Synthesis of 4-(4-methoxyphenoxy)-N-(4-morpholinophenyl)-5-nitropyrimidin-2-amine (3)***. A clean and efficient reported reaction condition was employed [22], where 4-morpholinoaniline (5 mmol) was added to a solution of 2-chloro-4-(4-methoxyphenoxy)-5-nitropyrimidine (**2**, 5 mmol) dissolved in acetonitrile. The reaction was then stirred at room temperature overnight. The mixture was then evaporated *in vacuo*, washed with water, NaHCO_3_, and brine. The organic layer was dried over Na_2_SO_4_ and purified by flash column chromatography (EA:Hex, 1:1) to yield compound **3** as an orange solid. Orange solid, yield: 43%, ^1^H NMR (400 MHz, DMSO-*d*_6_): δ 10.61 (s, 1H), 9.13 (s, 1H), 7.22 (d, *J* = 9.0 Hz, 2H), 7.13 (d, *J* = 8.7 Hz, 2H), 7.08 (d, *J* = 8.9 Hz, 2H), 6.58 (d, *J* = 8.8 Hz, 2H), 3.85 (s, 3H), 3.72 (t, *J* = 4.9 Hz, 4H), 2.98 (t, *J* = 4.4 Hz, 4H). Reported [40].

***Synthesis of 4-(4-methoxyphenoxy)-N2-(4-morpholinophenyl)pyrimidine-2,5-diamine (4)***. A solution of 4-(4-methoxyphenoxy)-N-(4-morpholinophenyl)-5-nitropyrimidin-2-amine (**3**, 1 mmol) was prepared using 50 mL of a 10% MC/MeOH mixture as a solvent, followed by adding 0.1 mmol of Pd/C under nitrogen, and the mixture was then stirred under hydrogen overnight. The metal was then filtered using celite, and the filtrate was evaporated under reduced pressure to give compound **4**. Grey solid, yield: 69%, ^1^H NMR (400 MHz, DMSO-*d*_6_): δ 8.58 (s, 1H), 7.82 (s, 1H), 7.28 (d, *J* = 9.0 Hz, 2H), 7.14–7.17 (m, 2H), 7.02–7.05 (m, 2H), 6.64 (d, *J* = 9.0 Hz, 2H), 4.51 (s, 2H), 3.81 (s, 3H), 3.71 (t, *J* = 4.8 Hz, 4H), 2.93 (t, *J* = 4.7 Hz, 4H). Reported [40].

***General procedure of final amide derivatives 5a–5d***. A small flask containing 0.3 mmol of the pre-final amine (**4**) and DIPEA (0.3 mmol) dissolved in dichloromethane (DCM, 5 mL) was cooled to 0 °C, and an equivalent amount of the appropriate benzoyl chloride was added. The reaction mixture was allowed to warm to room temperature and stirred overnight. The reaction mixture was then evaporated *in vacuo* and purified by flash column chromatography (20–50% EA/Hex) to obtain the final amides **5a–5d**.

***N-(4-(4-Methoxyphenoxy)-2-((4-morpholinophenyl)amino)pyrimidin-5-yl)benzo[d]*[1,3]*dioxole-5-carboxamide (5a)***. Yellow solid, yield: 56%, mp: 205.9–206.9 °C, HPLC purity: 6.43 min, 95.12%, ^1^H NMR (400 MHz, CDCl_3_): δ 9.13 (s, 1H), 7.82 (s, 1H), 7.36 (dd, *J* = 8.1, 1.5 Hz, 1H), 7.32 (d, *J* = 1.4 Hz, 1H), 7.14 (d, *J* = 8.8 Hz, 2H), 7.03 (d, *J* = 8.9 Hz, 2H), 6.88 (d, *J* = 8.9 Hz, 2H), 6.85 (s, 1H), 6.80 (d, *J* = 8.0 Hz, 1H), 6.64 (d, *J* = 8.8 Hz, 2H), 5.97 (s, 2H), 3.76–3.78 (m, 7H), 2.97 (t, *J* = 4.6 Hz, 4H). ^13^C NMR (100 MHz, CDCl_3_) δ 164.58, 160.39, 157.39, 155.41, 150.87, 150.65, 148.26, 146.73, 145.60, 132.56, 128.38, 123.07, 121.92, 119.94, 116.51, 114.59, 112.89, 108.23, 107.76, 101.90, 66.98, 55.71, 50.16. HRMS (ESI) *m/z* calculated for C_17_H_17_N_3_O_3_ [M+H]^+^: 542.2040. Found: 542.2040.

***3,4-Dimethoxy-N-(4-(4-methoxyphenoxy)-2-((4-morpholinophenyl)amino)pyrimidin-5-yl)benzamide (5b)***. Yellow solid, yield: 73%, mp: 119.1–120.1 °C, HPLC purity: 6.24 min, 97.24%, ^1^H NMR (400 MHz, CDCl_3_) δ 9.26 (s, 1H), 8.01 (s, 1H), 7.57 (d, *J* = 1.7 Hz, 1H), 7.45 (dd, *J* = 8.3, 1.8 Hz, 1H), 7.25 (d, *J* = 8.8 Hz, 2H), 7.14 (d, *J* = 9.0 Hz, 2H), 6.99 (d, *J* = 9.0, 2H), 6.93 (d, *J* = 8.2 Hz, 2H), 6.75 (d, *J* = 8.8 Hz, 2H), 3.99 (s, 3H), 3.97 (s, 3H), 3.86–3.89 (m, 7H). ^13^C NMR (100 MHz, CDCl_3_) δ 164.94, 160.40, 157.38, 155.39, 152.29, 150.63, 149.31, 146.74, 145.63, 132.56, 126.83, 123.07, 119.97, 119.5, 116.51, 114.59, 112.98, 110.87, 110.40, 66.98, 56.14, 56.11, 50.16. HRMS (ESI) *m/z* calculated for C_17_H_17_N_3_O_3_ [M+H]^+^: 558.2353. Found: 558.2352.

***3,4,5-Trimethoxy-N-(4-(4-methoxyphenoxy)-2-((4-morpholinophenyl)amino)pyrimidin-5-yl)benzamide (5c)***. Yellow solid, yield: 69%, mp: 179.5–180.5 °C, ^1^H NMR (400 MHz, CDCl_3_) δ 9.21 (s, 1H), 7.94 (s, 1H), 7.25 (d, *J* = 8.8 Hz, 2H), 7.14–7.16 (m, 4H), 7.01–6.98 (m, 3H), 6.75 (d, *J* = 8.8 Hz, 2H), 3.96 (s, 6H), 3.93 (s, 3H), 3.86–3.89 (m, 7H). ^13^C NMR (100 MHz, CDCl_3_) δ 165.23, 160.57, 157.41, 155.59, 153.42, 150.99, 146.81, 145.60, 141.56, 132.45, 129.68, 123.04, 120.04, 116.49, 114.60, 112.67, 104.74, 66.98, 60.99, 56.50, 55.71, 50.13. HRMS (ESI) *m/z* calculated for C_17_H_17_N_3_O_3_ [M+H]^+^: 588.2458. Found: 588.2458.

***3,5-Diethoxy-N-(4-(4-methoxyphenoxy)-2-((4-morpholinophenyl)amino)pyrimidin-5-yl)benzamide (5d)***. Yellow solid, yield: 59%, mp: 113.1–114.1 °C, ^1^H NMR (400 MHz, CDCl_3_): δ 9.16 (s, 1H), 7.90 (s, 1H), 7.14 (d, *J* = 8.4 Hz, 2H), 7.02 (d, *J* = 8.5 Hz, 2H), 6.86–6.92 (m, 5H), 6.64 (d, *J* = 8.0 Hz, 2H), 6.54 (s, 1H), 3.97 (q, *J* = 6.7 Hz, 4H), 3.78 (s, 7H), 2.98 (s, 4H), 1.33 (t, *J* = 6.8 Hz, 6H). ^13^C NMR (100 MHz, CDCl_3_) δ 165.22, 160.38, 157.39, 155.45, 150.61, 146.74, 145.59, 136.30, 132.54, 123.10, 119.97, 116.50, 114.58, 112.84, 105.58, 104.72, 66.98, 63.91, 55.70, 50.15, 14.76. HRMS (ESI) *m/z* calculated for C_17_H_17_N_3_O_3_ [M+H]^+^: 586.2666. Found: 586.2665.

***General procedure of final amide derivatives 5e–5g***. The appropriate carboxylic acid (1.15 eq.) and HATU (1.15 eq.) were first dissolved in DMF and stirred for 10 min, DIPEA (2.5 eq.) was then added, and the mixture stirred for another 5 min. The pre-final amine was finally added, and the reaction mixture was microwaved at 120 °C for 1 h. The reaction mixture was then washed several times using ethyl acetate and brine. The organic layer was then dried over Na_2_SO_4_ and purified by flash column chromatography (20–50% EA/Hex) to afford the final amides **5e–5g**.

***2-(3,5-Dimethoxyphenyl)-N-(4-(4-methoxyphenoxy)-2-((4-morpholinophenyl)amino)pyrimidin-5-yl)acetamide (5e)***. Yellow solid, yield: 51%, mp: 152.7–153.7 °C, HPLC purity: 6.61 min, 98.92%, ^1^H NMR (400 MHz, CDCl_3_): δ 8.92 (s, 1H), 7.31 (s, 1H), 7.12 (d, *J* = 8.6 Hz, 2H), 6.95 (d, *J* = 8.8 Hz, 2H), 6.84 (d, *J* = 8.8 Hz, 2H), 6.81 (s, 1H), 6.63 (d, *J* = 8.3 Hz, 2H), 6.43 (s, 2H), 6.32 (s, 1H), 3.75–3.77 (m, 7H), 3.68 (s, 6H), 3.64 (s, 2H), 2.97 (s, 4H). ^13^C NMR (100 MHz, CDCl_3_) δ 168.82, 161.42, 160.35, 157.23, 155.63, 150.88, 146.77, 145.54, 136.466, 132.49, 122.80, 120.02, 116.49, 114.44, 112.36, 107.43, 99.72, 66.97, 55.68, 55.39, 50.14, 44.71. HRMS (ESI) *m/z* calculated for C_17_H_17_N_3_O_3_ [M+H]^+^: 572.2509. Found: 572.2509.

***N-(4-(4-Methoxyphenoxy)-2-((4-morpholinophenyl)amino)pyrimidin-5-yl)-2-nitroisonicotinamide(5f)***. Orange solid, yield: 62%, mp: 170–171 °C, ^1^H NMR (400 MHz, CDCl_3_): δ 9.16 (s, 1H), 8.84 (d, *J* = 3.8 Hz, 1H), 8.71 (s, 1H), 8.25 (s, 1H), 8.18 (s, 1H), 7.24 (d, *J* = 7.4 Hz, 2H), 7.13 (d, *J* = 8.1 Hz, 2H), 6.99–7.04 (m, 3H), 6.75 (d, *J* = 7.8 Hz, 2H), 3.88 (s, 7H), 3.09 (s, 4H). ^13^C NMR (100 MHz, CDCl_3_) δ 161.02, 160.81, 157.56, 157.37, 156.31, 151.51, 150.12, 147.11, 145.69, 145.23, 131.90, 126.72, 122.97, 120.35, 116.36, 115.67, 114.67, 111.41, 66.94, 55.71, 49.97, 31.60, 22.66, 14.13. HRMS (ESI) *m/z* calculated for C_17_H_17_N_3_O_3_ [M+H]^+^: 544.1945. Found: 544.1945.

***N-(4-(4-Methoxyphenoxy)-2-((4-morpholinophenyl)amino)pyrimidin-5-yl)-3-(methylthio)benzamide (5g)***. Yellow solid, yield: 54%, mp: 106–107 °C, HPLC purity: 6.87 min, 99.29%, ^1^H NMR (400 MHz, CDCl_3_): δ 9.26 (s, 1H), 8.03 (s, 1H), 7.83 (s, 1H), 7.63 (d, *J* = 7.0 Hz, 1H), 7.40–7.46 (m, 2H), 7.25 (d, *J* = 8.8 Hz, 2H), 7.14 (d, *J* = 8.9 Hz, 2H), 6.99–7.01 (m, 3H), 6.75 (d, *J* = 8.9 Hz, 2H), 3.86–3.89 (m, 7H), 3.07 (t, *J* = 4.72 Hz, 4H), 2.57 (s, 3H). ^13^C NMR (100 MHz, CDCl_3_) δ 164.99, 160.48, 157.40, 155.58, 150.83, 146.79, 145.57, 140.19, 134.92, 132.48, 129.75, 129.09, 125.07, 123.15, 123.07, 120.02, 116.49, 114.59, 112.68, 66.98, 55.71, 50.14, 15.64. HRMS (ESI) *m/z* calculated for C_17_H_17_N_3_O_3_ [M+H]^+^: 544.2019. Found: 544.2018.

### 2.2. Biological Evaluation

#### 2.2.1. In Vitro Kinase Inhibition Assay

The in vitro kinase inhibition assay was carried out by Reaction Biology Corp. (Reaction Biology Corp., Chester, PA, USA) Kinase HotSpot^SM^ service (http://www.reactionbiology.com, accessed on 15 January 2021), following the previously reported methods [16,40]. (For details, see Supplementary Material).

#### 2.2.2. In Vitro Antitumor Activity towards 60 Cancer Cell Lines

The antitumor assay was performed according to the protocol of the Drug Evaluation Branch, NCI, Bethesda [41]. A 48 h drug exposure protocol was adopted, and sulforhodamine B (SRB) assay was utilized to assess the cell growth and viability, as reported earlier [42,43].

#### 2.2.3. Molecular Modeling Study

Crystal structure of LCK (PDB ID: 3KMM), DAPK1 (PDB code: 4TXC), FMS (PDB ID: 6N33), and LYN (PDB ID: 2ZVA) were downloaded from the protein data bank (www.pdb.org, accessed on 20 March 2021). LCK, FMS, and DAPK1 structures are all respectively complexed with small molecule inhibitors. Protein structures were prepared using the protein preparation wizard of the Schrodinger 2020 suite of the package at the default setting and 7.4 pH value. All ligands were sketched using ChemDraw Professional 16.0, saved as structure data file format, and imported to Ligprep module. Ligprep module of Schrodinger was used for preparing all ligands and geometry optimization. Re-docking X-ray ligands confirmed the reproducibility of the docking program (data not shown). All minimized conformations of ligands were docked into their own respective binding site using Glide’s standard precision module and produced 10 poses for each ligand. The docking figures were produced using the Discovery Studio Client 2020 package. We selected the docked poses with more negative docking scores and significant interactions.

## 3. Results and Discussion

### 3.1. Chemical Synthesis

**Scheme 1:**
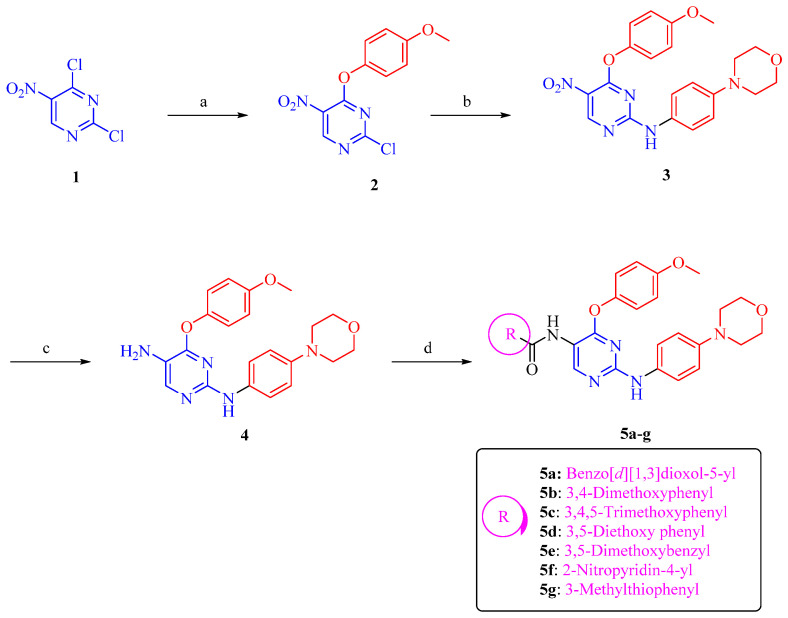
Reagents and conditions: (**a**) 4-Methoxyphenol, aq. NaHCO_3_, acetone, 0 °C to rt, 3 h; (**b**) 4-morpholinoaniline, MeCN, rt, overnight; (**c**) H_2_, 10% Pd/C, 10% DCM/methanol, rt, 12 h; (**d**) (i) for derivatives **5a–5d**: Appropriate acyl chloride, DIPEA, DCM, 0 °C to rt, overnight; (ii) for derivatives **5e–5g**: Appropriate carboxylic acid, DIPEA, HATU, DMF, MW, 120 °C, 1 h.

The newly synthesized target compounds (**5a–5g**) were prepared as outlined in Scheme 1. Starting from the commercially available 2,4-dichloro-5-nitropyrimidine (**1**). A solution of 4-methoxyphenol in a mixture of aqueous sodium bicarbonate and water was added to compound **1** in acetone to give compound **2** which was stirred with 4-morpholinoaniline in acetonitrile overnight to afford compound **3** as an orange solid. Compound **3** was reduced by stirring in a mixture of DCM/methanol (1:9) under hydrogen gas in the presence of a catalytic amount of palladium on carbon. The reduced pre-final amine (**4**) was then used to afford the final amide derivatives **5a–5g** either by stirring overnight with the corresponding benzoyl chloride in DCM solvent and DIPEA base to yield derivatives **5a–5d**, or by reacting it with the appropriate carboxylic acid in dimethylformamide and in the presence of HATU and DIPEA to afford compounds **5e–5g**. The structure elucidation and identification of the synthesized target hybrids were done with the aid of NMR and HRMS spectroscopy. The synthesis of compound **2** was confirmed through the presence of a signal corresponding to the methoxy group of the 4-methoxyphenoxy moiety at 3.81 ppm. ^1^H NMR chart of compounds **3** was characterized by the appearance of eight hydrogens attributable the morpholine ring and three hydrogens of the methoxy group of the 4-methoxyphenoxy moiety. The subsequent reduction of the nitro group to produce compound **4** was confirmed through the appearance of a new signal attributable to two new exchangeable protons of the newly generated amino group. The ^1^H NMR spectra of compounds **5a–5g** were all characterized by the presence of two peaks at 3.00–4.00 ppm attributable to the eight hydrogens of the morpholine ring and the three hydrogens of the methoxy group. In addition, the amide group of compounds **5a–5g** always displayed signals resonating in the range of 8.8–9.3 ppm of the ^1^H NMR spectra. Moreover, their ^13^C NMR spectra showed signals resonating in the range of 161–168 ppm characteristic to C = O carbons.

### 3.2. Biological Evaluation

#### 3.2.1. Assessment of Kinase Inhibitory Activity of Compound **5a** against a Panel of Kinases

As mentioned in the introduction, to get insights about the kinase inhibition profile of the hybrid small molecule **5a**, an in vitro screening over a panel of 14 cancer-related kinases was carried out. Accordingly, 10 µM concentrations of compound **5a** were used in a kinase inhibition assay over various kinase groups and families in the presence of 10 M ATP using HotSpotSM technology. To get a comprehensive picture of the inhibitory activities of the tested compound (**5a**) against the kinase panel, data are illustrated in Table 1. Interestingly, compound **5a** displayed promising inhibitory activities of more than 50% inhibition against four kinases: Colony-stimulating factor-1 receptor (FMS), lymphocyte-specific protein tyrosine kinase (LCK), tyrosine-protein kinase LYN, and death-associated protein kinase 1 (DAPK1) with inhibition values of 82.5 ± 0.6, 81.4 ± 0.6, 75.2 ± 0.0, and 55 ± 1.1%, respectively. Compound **5a** was also able to suppress the kinase activity of the epidermal growth factor receptor (EGFR), platelet-derived growth factor receptor-alpha (PDGFRα), and cyclin-dependent kinase 2 (CDK2) with modest inhibition values of 26.99 ± 0.9, 24.0 ± 0.4, and 20.1 ± 0.1%, respectively. Other kinases showed very little to no inhibition. Several studies have confirmed direct relationships between the most affected kinases (FMS, LCK, LYN, and DAPK1) and different human disorders including cancer [40,44,45,46,47,48,49]. Accordingly, these four kinases were selected to be included in further biological assays for the optimized hybrids **5b–5g** in a step to identify more active kinase inhibitors and to get structure-activity relationship (SAR) insights for this new marine-derived series.

#### 3.2.2. Assessment of Kinase Inhibitory Activity of Compounds **5b–5g** against FMS, LCK, LYN, and DAPK1 Kinases

The four protein kinases inhibited by the hybrid small molecule **5a** with more than 50% inhibition (FMS, LCK, LYN, and DAPK1) were selected to run an assessment for the optimized derivatives **5b–5g** at 10 µM concentration of each compound. Table 2 shows the percent inhibition values of the optimized compounds **5b–5g** over the four selected kinases in comparison to the results obtained for compound **5a**.

**Figure 2:**
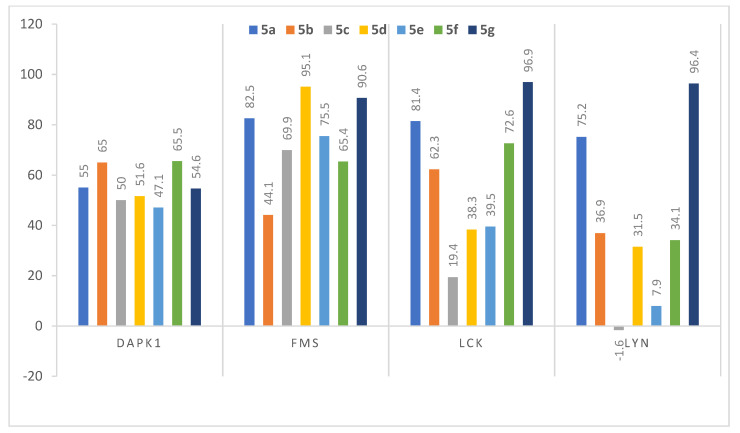
Schematic chart for percent enzyme inhibition (relative to DMSO controls) of all synthesized analogs at a concentration of 10 µM over DAPK1, FMS, LCK, and LYN kinases.

Replacement of the benzo[*d*][1,3]dioxole moiety in compound **5a** with 3,4,5-trimethoxyphenyl (**5c**) or 3,5-dimethoxyphenyl (**5e**) led to a noticeable decrease of the kinase inhibition against all four kinases (DAPK1, FMS, LCK, and LYN) with percent inhibition values of 50 ± 0.1, 69.9 ± 0.4, 19.4 ± 0.9, and −1.6 ± 0.3% for compound **5c** and 47.1 ± 0.7, 75.5 ± 0.8, 39.5 ± 0.4, and 7.9 ± 0.4% for compound **5e**, respectively. While compounds possessing 3,4-dimethoxyphenyl (**5b**) and 2-nitropyridin-4-yl (**5f**) showed a similar decrease pattern of the kinase inhibitory activity over FMS, LCK, and LYN kinases with percent inhibition values ranging from 34.1 ± 2.2 to 72.6 ± 0.6%, surprisingly, both compounds were able to elicit higher inhibitory activities against DAPK1 kinase compared to the parent hybrid compound **5a** with 65 ± 1.2 and 65.5 ± 1.4% inhibition, respectively. Interestingly, compound **5d** possessing 3,5-diethoxyphenyl moiety displayed the highest inhibitory activity over FMS kinase (95.1 ± 0.3% inhibition), while it demonstrated moderate inhibitory activities against DAPK1, LCK, and LYN kinases with 51.6 ± 0.5, 38.3 ± 4.2, and 31.5 ± 5.8%, respectively. As illustrated in Figure 2, the most broad-spectrum active compound in this series was compound **5g** possessing 3-methylthiophenyl moiety. While **5g** displayed a modest inhibitory activity against DAPK1 kinase with percent inhibition value of 54.6 ± 0.8%, it showed more than 90% inhibition against the other three tested kinases (90.6 ± 0.8, 96.9 ± 0.3, and 96.4 ± 0.1% over FMS, LCK, and LYN kinases, respectively). Based on these results, compounds **5d** and **5g** were subjected to further evaluation.

#### 3.2.3. Dose-Dependent Assay of the Most Active Analogs **5d** and **5g** over FMS, LCK, and LYN Kinases

Since only compounds **5d** and **5g** were able to inhibit FMS, LCK, and/or LYN kinases with percent inhibition of more than 90%, both compounds were selected for a further dose-dependent assay to determine their IC_50_ values over the corresponding kinases(s) in a 10-dose IC_50_ duplicate mode with a 3-fold serial dilution starting at 100 μM. The results were compared with the FDA-approved multiple kinase inhibitor imatinib [50]. As summarized in Table 3, compound **5d** was only assessed over FMS kinase where it demonstrated an IC_50_ value of 213 ± 1 nM, which is almost 5-fold more potent than imatinib. Compound **5g** was also able to show potent IC_50_ values of 110 ± 8, 87.7 ± 8.3, and 169 ± 31 nM against FMS, LCK, and LYN kinases, respectively. Compared to imatinib, compound **5g** was found to be ~ 9- and 2-fold more potent than imatinib over FMS and LCK kinases, respectively.

#### 3.2.4. Efficacy and Spectrum against Diverse Cancer Cells in Growth Inhibition (GI) Assays

**Figure 3:**
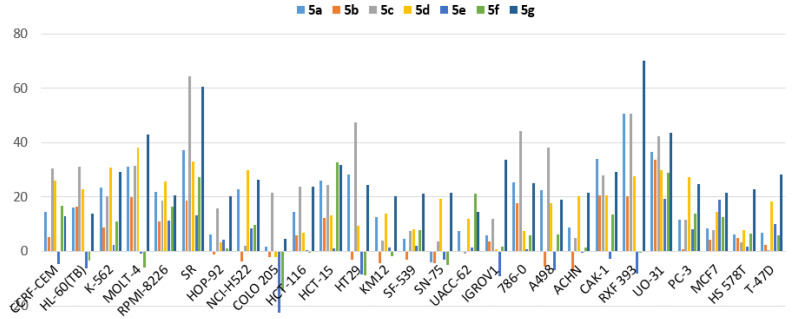
Schematic chart of percent inhibition of all synthesized compounds **5a–5g** against a panel of cancer cell lines.

The inhibition results of tumor cell growth by the newly synthesized compounds (**5a–5g**) are described in Table 4. The reported measurements have been performed at the NIH National Cancer Institute, USA by a standardized assay including a panel of 60 different tumor cell lines (Supplementary Data) [51]. The following cancer cell types were included in these assays: Leukemia, non-small cell lung cancer, colon cancer, CNS cancer, melanoma, ovarian cancer, renal cancer, prostate, and breast cancer. The data provided in Table 4, as well as the graphical representation of the inhibitory activity of the synthesized compounds on the different cell lines (Figure 3), revealed that the dimethoxy substitution of the phenyl ring reduced the anti-cancer activity, as in the 3,5-dimethoxy substituted compound (**5e**) which totally lost the inhibitory activity. Additionally, the 3,4-dimethoxy substituted compound (**5b**) also suffered poor activity against the cancer cell lines. Replacing the dimethoxy substitutions with a 3,5-diethoxy substitution (**5d**) significantly increased the inhibitory activity, while the incorporation of an ortho-substituted nitro group on the phenyl ring did not cause a significant improvement of the activity. While the parent marine-derived compound **5a** was only able to inhibit the RXF 393 cell line of renal cancer with 50.5% growth inhibition, both derivatives **5c** and **5g** showed a significant antiproliferative activity against the SR cell line of leukemia (64.4 and 60.5% growth inhibition, respectively) as well as the RXF 393 cell line of renal cancer with 50.6 and 70.1% growth inhibition, respectively. The other synthesized analogs **5b**, **5d**, **5e**, and **5f** exhibited moderate to poor inhibitory effects on the different cancer cell lines.

#### 3.2.5. Molecular Docking Studies

A molecular docking study was performed on the active binding regions of LCK, FMS, DAPK1, and LYN proteins. This study was conducted to provide a deeper view of how the changes of the functional groups may affect the activity of the compounds. The docking models over each enzyme are discussed separately in the following subsections.

##### Molecular Docking Models within the LCK Binding Site

**Figure 4:**
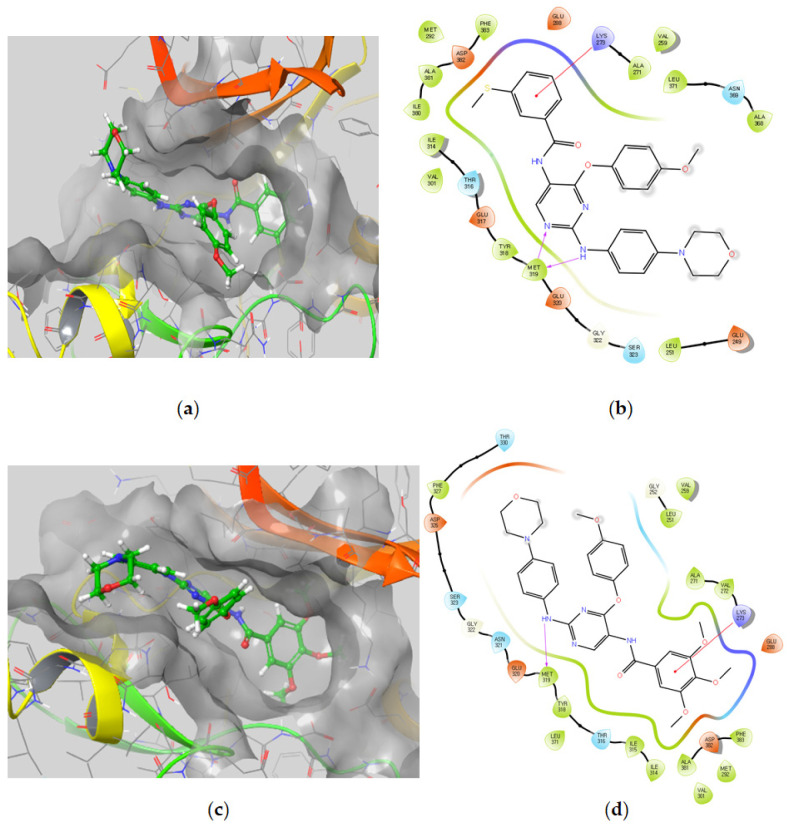
Docked complexes of compounds **5g** and **5c** with LCK. (**a**,**c**) 3D docking models of compounds **5g** and **5c** into LCK binding pocket, respectively. (**b**,**d**) 2D interaction diagrams of compounds **5g** and **5c** with LCK, respectively.

As shown in Table 5, the docked poses of all synthesized compounds showed a direct correlation between the predicted binding affinity of the tested compounds to the active site and their respective LCK inhibition. Compounds with the highest docking scores **5a** (−9.75), **5f** (−9.62), and **5g** (−9.32) correspondingly demonstrated the highest LCK inhibition (81.4, 72.6, and 96.9%, respectively). On the contrary, compounds with lower docking scores **5c** (−7.39) and **5d** (−6.844) exhibited only 19.4 and 38.3% inhibition of LCK, respectively. The inhibitory activity of compounds **5a**, **5b**, **5f**, and **5g** against LCK could be explained due to their ability to establish a minimum of two hydrogen bonds with Met319. Compound **5c**, on the other hand, was able to establish only one hydrogen bond with Met319 in addition to a weak π–π stacking with the pyridine ring, which explains the reason for its weak binding affinity to the binding site of the LCK receptor leading to a weak inhibitory activity. The binding mode of compound **5g** that possesses the highest LCK effect is compared to that of the least active compound **5c** in Figure 4.

##### Molecular Docking Models within the FMS Binding Site

**Figure 5:**
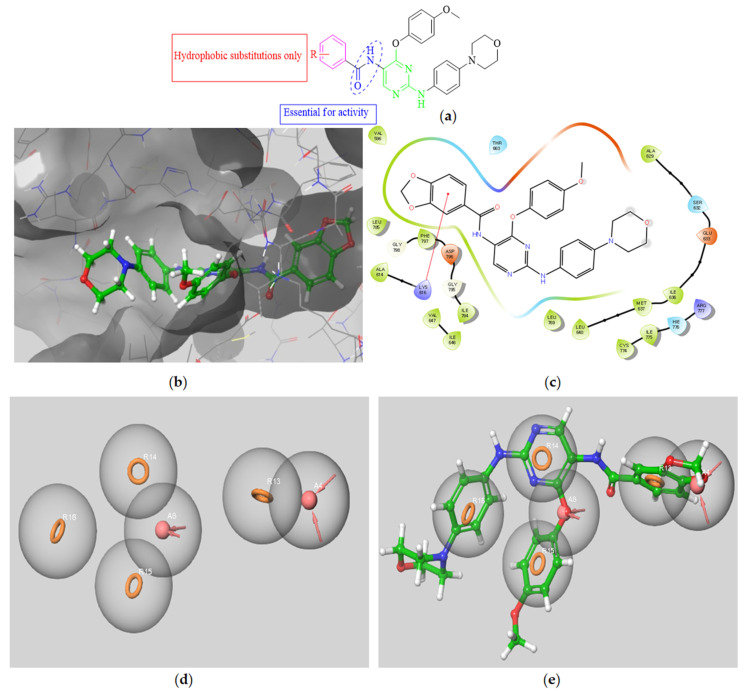
(**a**) General SAR of the synthesized compounds against FMS kinase. (**b**) 3D docking model of compound **5a** into the FMS pocket. (**c**) 2D interaction diagram of compound **5a** with FMS. (**d**) predicted hypothesis of the essential binding interactions using compound **5a** as a reference. (**e**) compound **5a** overlaid on the generated e-pharmacophore hypothesis.

Compounds **5a**, **5b**, **5f**, and **5g** had the highest docking scores of −7.57, −6.81, −6.38, and −6.23, respectively, while compounds **5c**, **5d**, and **5e** demonstrated comparatively weaker docking scores of −3.26, −4.47, and −5.63, respectively. Despite the relative difference of these docking scores, several characteristics were elucidated through the docking study. One such feature is the amide group responsible for establishing a hydrogen bond between the amide group of the ligand and GLU633. Thus, the amide group was found to be essential for FMS inhibitory activity. To understand the difference in binding activity and identify the important binding groups, an energy-optimized pharmacophore (e-pharmacophore) hypothesis using “Develop a Pharmacophore from Receptor Cavity” option in the phase module was developed. Six pharmacophore sites were predicted, and the final hypothesis consisted of four aromatic rings (R13, 14, 15, and R16) and two H-bond acceptors (A8 and A4) as shown in Figure 5. The SAR diagram of the synthesized compounds, the top score docking model of compound **5a**, and its e-pharmacophore hypothesis are illustrated in Figure 5.

##### Molecular Docking Models within the DAPK1 Binding Site

**Figure 6:**
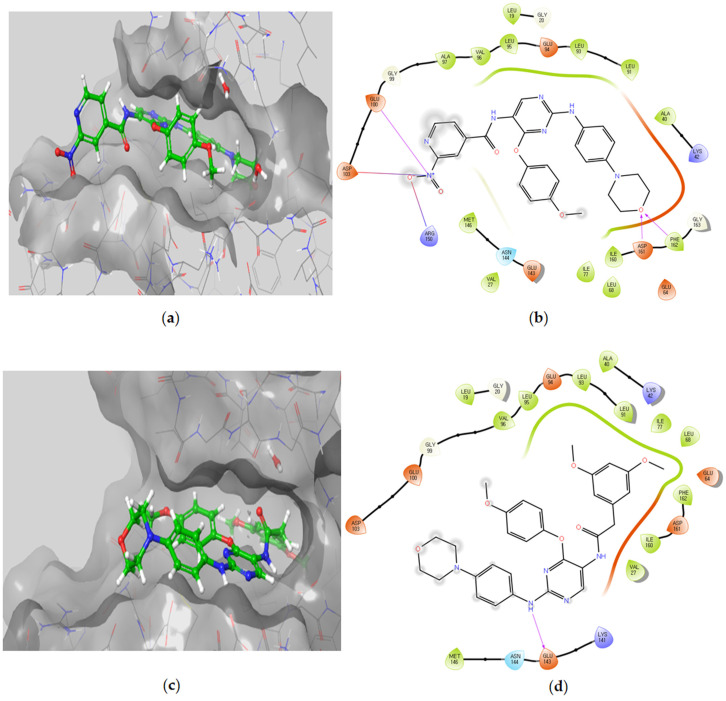
Docked complexes of compounds **5f** and **5e** with DAPK1. (**a**,**c**) 3D docking models of compounds **5f** and **5e** into DAPK1 binding pocket, respectively. (**b**,**d**) 2D interaction diagrams of compounds **5f** and **5e** with DAPK1, respectively.

All synthesized compounds displayed almost similar inhibitory activity over DAPK1, with a range of percent inhibition varying from 65.5% (**5f**) to 47.1% (**5e**). This difference in activity could be explained due to the difference of their binding modes to the active site residue. Compound **5f** formed one salt bridge and four hydrogen bonds, two of these hydrogen bonds were formed via the oxygen of the morpholine ring with Asp161 and Phe162, while the other two hydrogen bonds were established via the nitro group which acted as a hydrogen bond acceptor for Glu100 and Asp103. On the other hand, the least active compound **5e** was only able to form one hydrogen bond through the NH of the morpholino moiety with GLU143. The 2D predicted interaction diagram comparing both compounds **5f** and **5e** is demonstrated in Figure 6.

##### Molecular Docking Models within the LYN Binding Site

**Figure 7:**
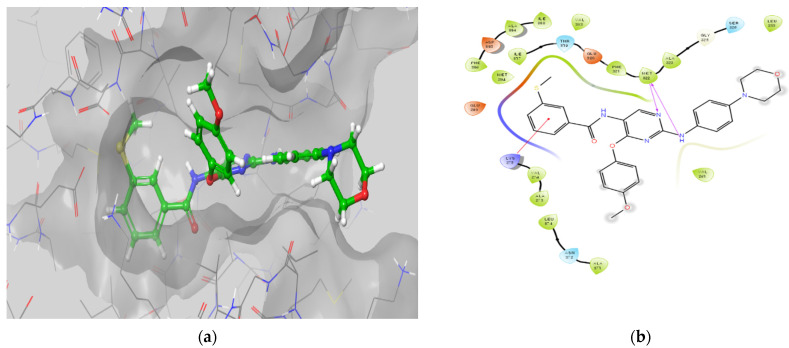
Docked complex of compound **5g** with LYN. (**a**) 3D docking model of compound **5g** into LYN binding pocket. (**b**) 2D interaction diagram of compound **5g** with LYN.

Among the synthesized compounds, only compound **5g** showed high activity against the LYN kinase with 96.4% inhibition at a single dose concentration of 10 µM, while the other synthesized compounds exhibited moderate to weak activity with compound **5c** being inactive (−1.6% inhibition). Through molecular docking, compound **5g** which has a strong binding affinity to the active site residue of the LYN protein (docking score of −9.979) was able to form two hydrogen bonds and one π–π interaction with the receptor active site, all within a distance of less than 3.5 Å. Conversely, compound **5c** exhibited a much weaker docking score of −5.94. The other synthesized compounds displayed moderate binding scores ranging from −6.4 to −7.9, which explains their relatively weak activity as LYN inhibitors. The predicted interaction of the most active compound **5g** with the LYN kinase is illustrated in Figure 7.

#### 3.2.6. In Silico Pharmacokinetic Study

Pharmacokinetic properties such as absorption, metabolism, excretion, and toxicity (ADMET) play a vital role in developing active therapeutic agents. A good antagonistic interaction of inhibitors with a receptor protein or enzyme does not warrant the capability of an inhibitor as a drug. One of the foremost causes of drug candidates to fail in their clinical experiments is the possession of poor ADME characteristics and unfavorable toxicology [52]. Subsequently, ADME analysis is crucial in drug development [53]. Hence, the pharmacokinetic properties of the final targeted compounds were predicted using the freely accessible web server of SwissADME (a machine learning platform used to predict small-molecule pharmacokinetic properties relying on distance/pharmacophore patterns encoded as graph-based signatures) [54] (see Supplementary File for more details). ADME is based on Lipinski’s rule of five and assists in the approval of inhibitors for biological systems. Apart from efficacy and toxicity, various drug development failures are due to poor pharmacokinetics and bioavailability [55]. Gastrointestinal absorption and brain access are two pharmacokinetic behaviors crucial to be estimated at various stages of the drug discovery processes [56]. All the synthesized compounds were subjected to an in silico pharmacokinetic study (Table 6).

The polar surface area (PSA) or topological polar surface area (TPSA) is characterized as the surface sum over every polar atom or molecule, predominantly oxygen and nitrogen, comprising their attached hydrogen atoms. PSA is frequently used as a medicinal chemistry metric for enhancing the drug’s capability to permeate cells. Molecules with a polar surface area of higher than 140 Å² are likely to be inadequate at permeating cell membranes. For a molecule to possess the ability to infiltrate BBB (and thereby be able to exert its effects on the receptors of the central nervous system), a PSA less than 90 angstroms squared is usually considered necessary [57]. Accordingly, among the synthesized compounds, only compound **5f** (TPSA of 156.2 Å²) is predicted to be unable to penetrate the cellular membrane easily.

On the other hand, compounds **5a–5e** and **5g** were found to possess appropriate TPSA values (higher than 90 and below 140 Å²) predicting their ability to penetrate the cells and exert their effects without any possible CNS side effects. Nevertheless, all the synthesized compounds were predicted to suffer from poor to moderate solubility. This, coupled with the fact that compounds **5c**, **5d**, **5f**, and **5g** were predicted to have low intestinal absorption. This means that further future modifications of the structures should be carried out to improve the oral bioavailability for this series and maximize their effectiveness.

## 4. Conclusions

A new series of hybrid small molecules (**5a–5g**) was developed based on chemical moieties originating from two marine natural products (Meridianin E and Leucettamine B). A single dosage of 10 µM of the parent hybrid **5a**, which contains the benzo[*d*][1,3]dioxole moiety of Leucettamine B, inhibited the activity of FMS, LCK, LYN, and DAPK1 kinases by 82.5 ± 0.6, 81.4 ± 0.6, 75.2 ± 0.0, and 55 ± 1.1%, respectively. Further optimizations led to compound **5g** (the most potent multi-kinase inhibitor of this new series) with IC_50_ values of 110, 87.7, and 169 nM against FMS, LCK, and LYN kinases, respectively, which is 9- and 2-fold more potent than the multi-kinase inhibitor imatinib over both FMS and LCK kinases, respectively. Compound **5g** also showed promising antitumor activities against leukemia SR and renal RXF 393 cell lines with 60 and 70% inhibition. Supported by the computational studies including docking and ADME simulations, compound **5g** is reported as a promising marine-derived multi-kinase potent inhibitor worthy of further investigation.

## Figures and Tables

**Table 1:** In vitro inhibition screening results of compound **5a** against a panel of 17 kinases at a single dose of 10 µM.

Type of Kinase	Family	Kinase	Percent Inhibition
Receptor Tyrosine Kinases	TAM family	c-MER	4.4 ± 0.1
	EGF receptor family	EGFR	26.99 ± 0.9
	PVR family	FMS	82.5 ± 0.6
		PDGFRα	24.0 ± 0.4
		FLT1/VEGFR1	−2.4 ± 1.7
		KDR/VEGFR2	7.9 ± 1.4
	HGF receptor	c-MET	12.0 ± 13.7
Non-Receptor Tyrosine Kinases	SRC-B family	LCK	81.4 ± 0.6
		LYN	75.2 ± 0.0
	JAK family	JAK3	11.2 ± 1.2
Tyrosine Kinase-Like kinases	RAF family	BRAF	4.1 ± 10.5
Calcium/Calmodulin-dependent kinases (CAMKs)	DAPK family	DAPK1	55 ± 1.1
CMGC serine/threonine kinases	Cyclin-dependent kinase family	CDK2/cyclin A	20.1 ± 0.1
P21-activated serine/threonine kinases	PAK Family	PAK1	−29.1 ± 3.4

**Table 2:** Percent inhibition values of the synthesized compounds **5a–5g** over the selected kinases at a single dose concentration of 10 µM.

Cpd	Chemical Structure	Percent Inhibition ^a^			
		DAPK1	FMS	LCK	LYN
**5a**	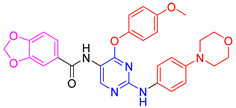	55 ± 1.1	82.5 ± 0.6	81.4 ± 0.6	75.2 ± 0.0
**5b**	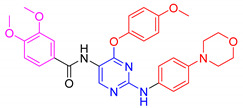	65 ± 1.2	44.1 ± 0.2	62.3 ± 0.8	36.9 ± 4.5
**5c**	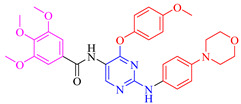	50 ± 0.1	69.9 ± 0.4	19.4 ± 0.9	−1.6 ± 0.3
**5d**	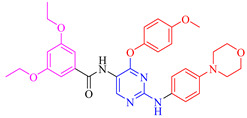	51.6 ± 0.5	95.1 ± 0.3	38.3 ± 4.2	31.5 ± 5.8
**5e**	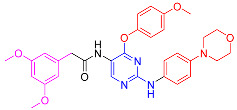	47.1 ± 0.7	75.5 ± 0.8	39.5 ± 0.4	7.9 ± 0.4
**5f**	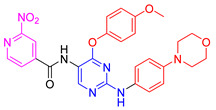	65.5 ± 1.4	65.4 ± 0.1	72.6 ± 0.6	34.1 ± 2.2
**5g**	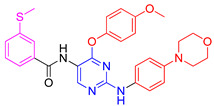	54.6 ± 0.8	90.6 ± 0.8	96.9 ± 0.3	96.4 ± 0.1

^a^ Percent inhibition values of different kinases at a single dose of 10 µM of the prepared compound.

**Table 3:** IC_50_ of most active compounds **5d** and **5g**.

Compound	FMS IC_50_ (nM)	LCK IC_50_ (nM)	LYN IC_50_ (nM)
**5d**	213 ± 1	NT	NT
**5g**	110 ± 8	87.7 ± 8.3	169 ± 31
Imatinib	1000	160	190

**Table 4:** The growth inhibition percentages of the synthesized compounds over the most sensitive cell lines at a single dose concentration of 10 µM.

Cancer Type	Cell Line	Percent Growth Inhibition (GI)						
		5a	5b	5c	5d	5e	5f	5g
Leukemia	CCRF-CEM	14.46	5.37	30.5	26.1	−4.73	16.66	12.96
	HL-60(TB)	16.16	16.41	31.0	22.8	−6.34	−3.19	13.94
	K-562	23.4	8.76	20.4	30.7	2.31	10.89	29.1
	MOLT-4	31.1	19.9	31.6	38.2	−0.81	−5.98	43.1
	RPMI-8226	21.8	11.12	18.76	25.6	11.31	16.32	20.5
	SR	37.1	18.75	64.4	33.2	13.21	27.4	60.5
Non-Small Cell Lung Cancer	HOP-92	6.16	−1.19	15.76	3.34	4.46	1.12	20.2
	NCI-H522	22.7	−3.5	2.25	29.8	8.4	9.77	26.4
Colon Cancer	COLO 205	1.75	−2.21	21.6	−2.11	−22.42	−14.56	4.7
	HCT-116	14.57	6.01	23.9	6.73	0.34	−0.19	23.7
	HCT-15	26.0	12.32	34.4	13.24	1.13	32.8	31.7
	HT29	28.3	−2.93	47.4	9.54	−8.47	−8.86	24.3
	KM12	12.72	−4.34	3.99	14	1.32	−1.85	20.2
CNS Cancer	SF-539	4.73	−3.17	7.64	8.01	2.09	7.72	21.1
	SNB-75	−3.98	−4.29	3.59	19.2	−3.1	−4.975	21.5
Melanoma	UACC-62	7.38	0.1	−0.63	11.99	1.34	21.4	14.69
Ovarian Cancer	IGROV1	5.92	3.6	11.87	0.72	−9.02	1.91	33.9
Renal Cancer	786-0	25.3	17.64	44.2	7.41	0.75	5.78	25.0
	A498	22.4	−5.56	38.3	17.86	−6.85	6.39	18.93
	ACHN	8.91	−7.28	4.83	20.4	−0.26	1.61	21.4
	CAKI-1	33.9	20.6	28.1	20.6	−2.58	13.69	29.4
	RXF 393	50.5	20.4	50.6	27.5	−8.16	−0.36	70.1
	UO-31	36.7	33.6	42.3	29.9	19.36	29	43.7
Prostate Cancer	PC-3	11.71	0.75	11.58	27.3	8.05	13.85	24.9
Breast Cancer	MCF7	8.51	4.24	7.94	14.43	18.87	12.54	21.5
	HS 578T	6.26	5.09	3.23	7.96	1.64	6.7	22.7
	T-47D	6.78	2.41	0.52	18.4	10.16	5.93	28.2

**Table 5:** Computational analysis of all synthesized compounds against LCK.

Compound	Docking Score	Ligand Atoms	Receptor Atoms	Interaction Type	Percent Inhibition
**5a**	−9.75	N3 N7 O38	Met319 Met319 Asp382	HBA HBD HBA	81.4 ± 0.6
**5b**	−8.15	N3 N7	Met319 Met319	HBA HBD	62.3 ± 0.8
**5c**	−7.39	N7 Pyridine ring	Met319 Lys273	HBD π-π stacking	19.4 ± 1.0
**5d**	−6.84	O31 O27	Ser329 Asp382	HBA HBA	38.3 ± 4.2
**5e**	−8.96	O31 O27	Met319 SER323	HBA HBA	37.5 ± 0.3
**5f**	−9.62	N3 N7 Pyridine ring N38 O39 O40	Met319 Met319 Lys273 Glu288 Phe383 Asp382	HBA HBD π-π stacking Salt bridge HBA HBA	72.6 ± 0.6
**5g**	−9.32	N3 N7 Pyridine ring	Met319 Met319 Lys273	HBA HBD π-π stacking	96.9 ± 0.3

**Table 6:** Predicted pharmacokinetic properties of compounds **5a–5g**.

Compound	TPSA	Solubility in Water	BBB Permeability	Intestinal Absorption
**5a**	116.3	Moderately soluble	no	high
**5b**	116.3	Moderately soluble	no	high
**5c**	125.5	Moderately soluble	no	low
**5d**	116.3	Poorly soluble	no	low
**5e**	116.3	Moderately soluble	no	high
**5f**	156.5	Moderately soluble	no	low
**5g**	123.1	Poorly soluble	no	low

## Supplementary Materials

The following are available online at https://www.mdpi.com/article/10.3390/biomedicines9091131/s1. General methods and instruments of Chemistry; charts of NMR, HPLC, and HRMS; biology protocols and raw data for kinase inhibition IC_50_ determination; original anticancer data obtained from NCI (USA) and Swiss ADME.
