# Supplementary material for: Development of New Meridianin/Leucettine-Derived Hybrid Small Molecules as Nanomolar Multi-Kinase Inhibitors with Antitumor Activity
Source: Biomedicines. 2021 Sep 1;9(9):1131. doi: 10.3390/biomedicines9091131 (PMC8468039; doi:10.3390/biomedicines9091131)
Supplement: Supplementary file 1 [file biomedicines-09-01131-s001.zip › biomedicines-1272627-Supplementary.pdf]

biomedicines

Supplementary material

Development of New Meridianin/Leucettine-Derived Hybrid Small Molecules as Nanomolar Multi-Kinase Inhibitors with Antitumor Activity

Mohamed H. Elsherbeny [1,2,3], Ahmed Elkamhawy [4,5,*], Hossam Nada [4,6], Magda H. Abdellattif [7], Kyeong Lee [4] and Eun Joo Roh [1,2,*]

[1] Chemical Kinomics Research Center, Korea Institute of Science and Technology (KIST), Seoul 02792, Korea
[2] Division of Bio-Medical Science & Technology, KIST School, University of Science and Technology, Seoul 02792, Korea
[3] Pharmaceutical Chemistry Department, Faculty of Pharmacy, Ahram Canadian University, Giza 12566, Egypt; mohamed.alsherbeny@pharma.asu.edu.eg (M.H.E.)
[4] College of Pharmacy, Dongguk University-Seoul, Goyang 10326, Korea; hossam_hammouda@dgu.ac.kr (H.N.); kaylee@dongguk.edu (K.L.)
[5] Department of Pharmaceutical Organic Chemistry, Faculty of Pharmacy, Mansoura University, Mansoura 35516, Egypt
[6] Pharmaceutical Chemistry Department, Faculty of Pharmacy, Badr University, Cairo 11829, Egypt
[7] Department of Chemistry, College of Science, Taif University, P.O. Box 11099, Taif 21944, Saudi Arabia; m.hasan@tu.edu.sa (M.H.A)
* Correspondence: a_elkamhawy@mans.edu.eg; a.elkamhawy@dongguk.edu (A.E.); r8636@kist.re.kr (E.J.R.)

Contents

1. **General methods and instruments (Chemistry)**
2. **^{1}H NMR and ^{13}C NMR data of compounds 5a–g.**
3. **LC and MS conditions.**
4. **Representative purity and HRMS data of compounds 5a–g.**
5. **Protocol of Kinase assay**
6. **Biology raw data for IC_{50} determination.**
7. **Antitumor data sheets carried out at NCI, USA.**
8. **ADME (SwissADME)**

1. General methods and instruments (Chemistry)

The NMR spectra were obtained on Bruker Avance 400. ^1H NMR spectra were referenced to tetramethylsilane (δ = 0.00 ppm) as an internal standard and are reported as follows: chemical shift, multiplicity (br = broad, s = singlet, d = doublet, t = triplet, dd = doublet of doublet, m = multiplet). Column chromatography was performed on Merck Silica Gel 60 (230−400 mesh) and eluting solvents for all these chromatographic methods are noted as appropriated-mixed solvent with given volume-to-volume ratios. TLC was carried out using glass sheets pre-coated with silica gel 60 F_{254} purchased by Merk. The purity of samples was determined by analytical HPLC using a Water ACQUITY UPLC (CORTECS^{TM}) with C18 column (2.1 mm x 100 mm; 1.6 μm) at temperature 40 °C. HPLC data were recorded using parameters as follows: 0.1% formic acid in water and 0.1% formic acid in methanol and flow rate of 0.3 mL/min. High-resolution spectra were performed on Waters ACQUITY UPLC BEH C18 1.7μ−Q-TOF SYNAPT G2-Si High-Definition Mass Spectrometry.

2. ^{1}H NMR and ^{13}C NMR data of compounds 5a–g

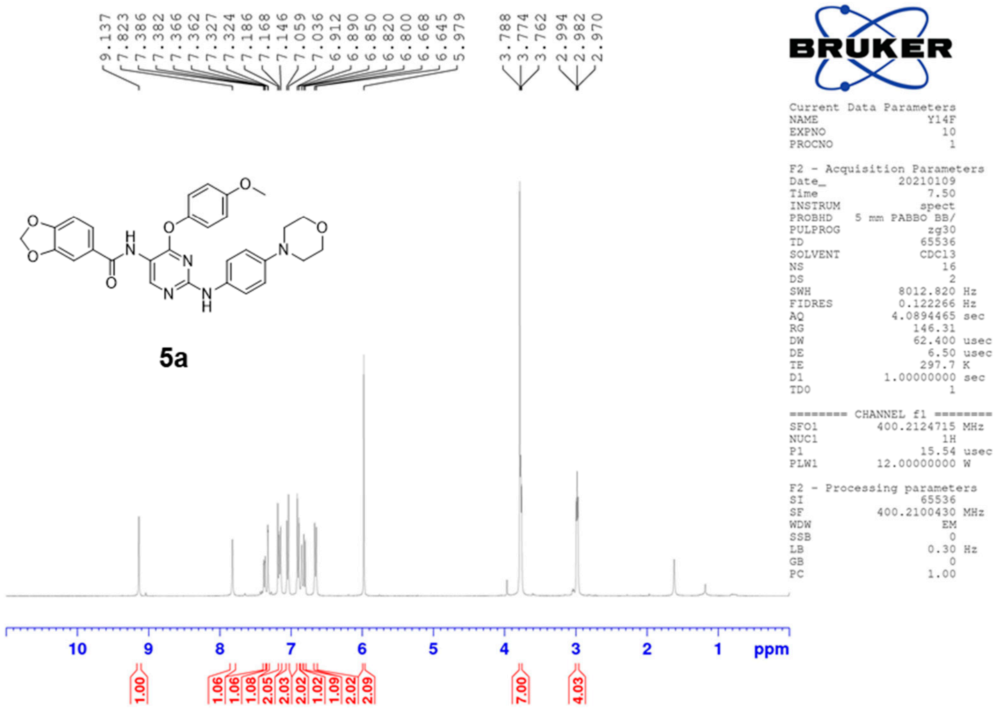

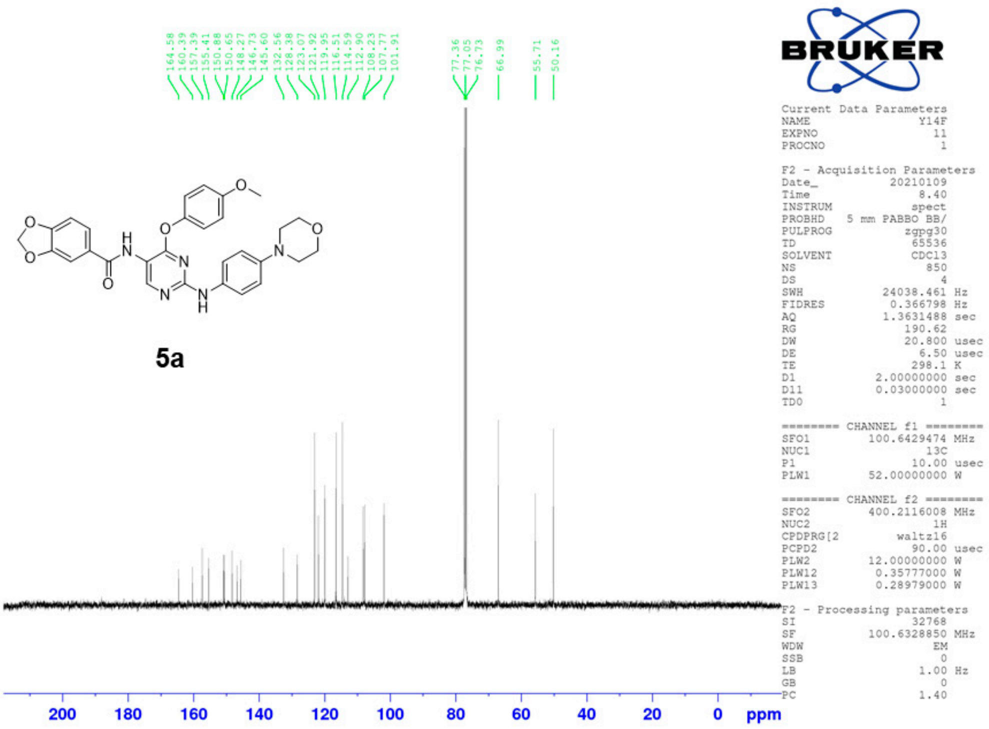
164.58 160.39 157.39 155.41 150.88 150.65 148.27 146.73 145.60 132.56 128.38 123.07 121.92 119.95 116.51 114.59 112.90 108.23 107.77 101.91
77.36 77.05 76.73
66.99
55.71
50.16
5a
BRUKER
Current Data Parameters
NAME Y14F
EXPNO 11
PROCNO 1
F2 - Acquisition Parameters
Date_ 20210109
Time 8.40
INSTRUM spect
PROBHD 5 mm PABBO BB/
PULPROG zgpg30
TD 65536
SOLVENT CDCl3
NS 850
DS 4
SWH 24038.461 Hz
FIDRES 0.366798 Hz
AQ 1.3631488 sec
RG 190.62
DW 20.800 usec
DE 6.50 usec
TE 298.1 K
D1 2.00000000 sec
D11 0.03000000 sec
TD0 1
======== CHANNEL f1 ========
SFO1 100.6429474 MHz
NUC1 13C
P1 10.00 usec
PLW1 52.00000000 W
======== CHANNEL f2 ========
SFO2 400.2116008 MHz
NUC2 1H
CPDPRG[2 waltz16
PCPD2 90.00 usec
PLW2 12.00000000 W
PLW12 0.35777000 W
PLW13 0.28979000 W
F2 - Processing parameters
SI 32768
SF 100.6328850 MHz
WDW EM
SSB 0
LB 1.00 Hz
GB 0
PC 1.40
200 180 160 140 120 100 80 60 40 20 0 ppm

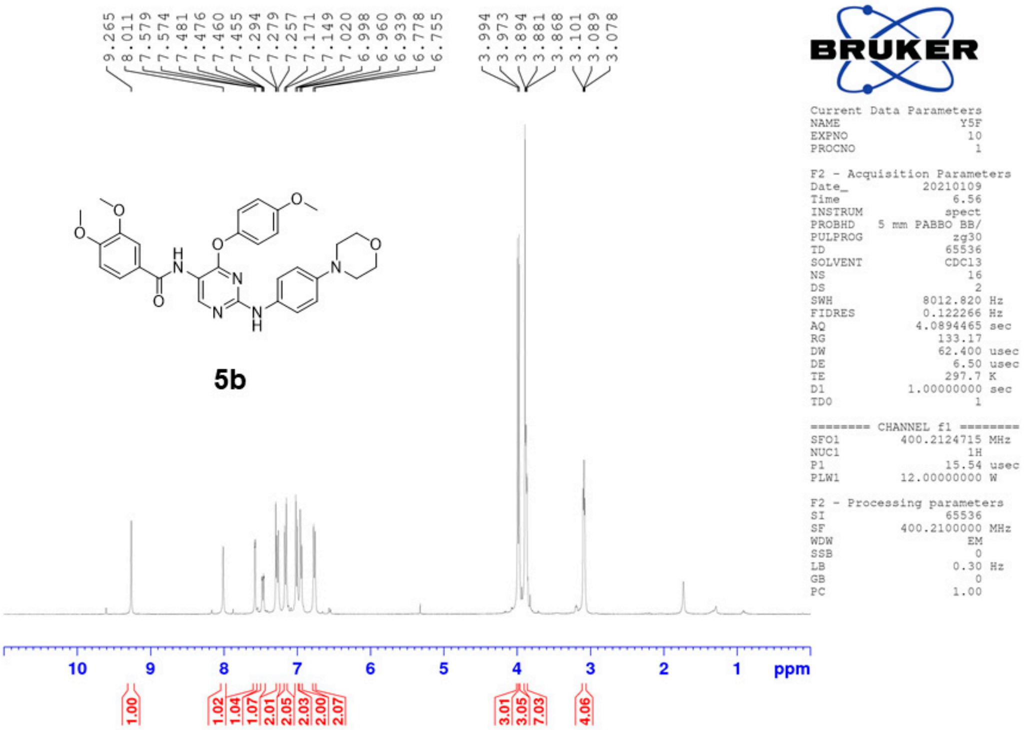
9.265
8.011
7.579
7.574
7.481
7.476
7.460
7.455
7.294
7.279
7.257
7.171
7.149
7.020
6.998
6.960
6.939
6.778
6.755
3.994
3.973
3.894
3.881
3.868
3.101
3.089
3.078
5b
Current Data Parameters
NAME Y5F
EXPNO 10
PROCNO 1
F2 - Acquisition Parameters
Date_ 20210109
Time 6.56
INSTRUM spect
PROBHD 5 mm PABBO BB/
PULPROG zg30
TD 65536
SOLVENT CDCl3
NS 16
DS 2
SWH 8012.820 Hz
FIDRES 0.122266 Hz
AQ 4.0894465 sec
RG 133.17
DW 62.400 usec
DE 6.50 usec
TE 297.7 K
D1 1.00000000 sec
TD0 1
======== CHANNEL f1 ========
SFO1 400.2124715 MHz
NUC1 1H
P1 15.54 usec
PLW1 12.00000000 W
F2 - Processing parameters
SI 65536
SF 400.2100000 MHz
WDW EM
SSB 0
LB 0.30 Hz
GB 0
PC 1.00
10 9 8 7 6 5 4 3 2 1 ppm
1.00
1.02
1.04
1.07
2.01
2.05
2.03
2.00
2.07
3.01
3.05
7.03
4.06

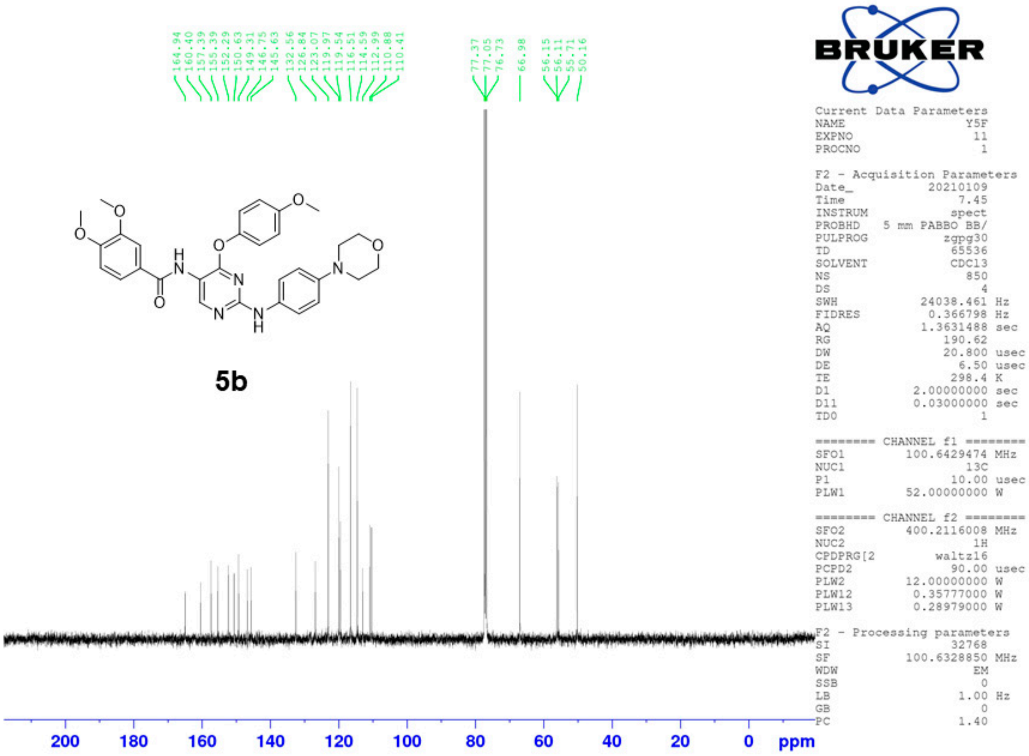
164.94
160.40
157.39
155.39
152.29
150.63
149.31
146.75
145.63
132.56
126.84
123.07
119.97
119.54
116.51
114.59
112.99
110.88
110.41
77.37
77.05
76.73
66.98
56.15
56.11
55.71
50.16
5b
Current Data Parameters
NAME Y5F
EXPNO 11
PROCNO 1
F2 - Acquisition Parameters
Date_ 20210109
Time 7.45
INSTRUM spect
PROBHD 5 mm PABBO BB/
PULPROG zgpg30
TD 65536
SOLVENT CDCl3
NS 850
DS 4
SWH 24038.461 Hz
FIDRES 0.366798 Hz
AQ 1.3631488 sec
RG 190.62
DW 20.800 usec
DE 6.50 usec
TE 298.4 K
D1 2.00000000 sec
D11 0.03000000 sec
TD0 1
======== CHANNEL f1 ========
SFO1 100.6429474 MHz
NUC1 13C
P1 10.00 usec
PLW1 52.00000000 W
======== CHANNEL f2 ========
SFO2 400.2116008 MHz
NUC2 1H
CPDPRG[2 waltz16
PCPD2 90.00 usec
PLW2 12.00000000 W
PLW12 0.35777000 W
PLW13 0.28979000 W
F2 - Processing parameters
SI 32768
SF 100.6328850 MHz
WDW EM
SSB 0
LB 1.00 Hz
GB 0
PC 1.40
200 180 160 140 120 100 80 60 40 20 0 ppm

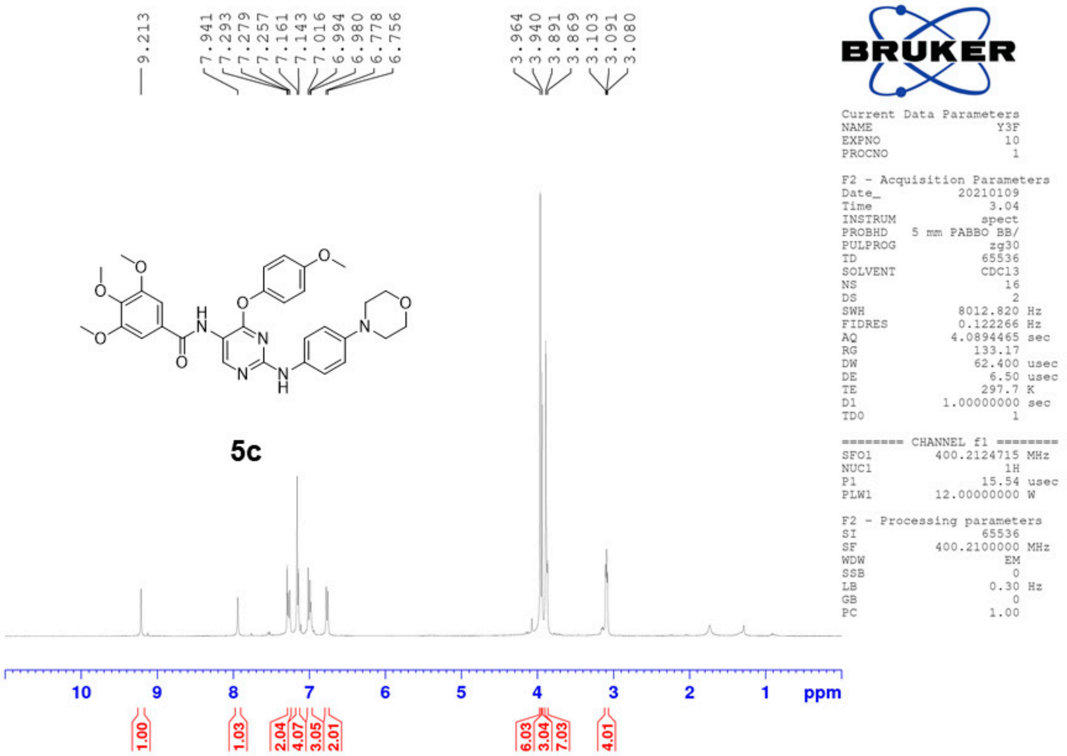
9.213
7.941
7.293
7.279
7.257
7.161
7.143
7.016
6.994
6.980
6.778
6.756
3.964
3.940
3.891
3.869
3.103
3.091
3.080
BRUKER
Current Data Parameters
NAME Y3F
EXPNO 10
PROCNO 1
F2 - Acquisition Parameters
Date_ 20210109
Time 3.04
INSTRUM spect
PROBHD 5 mm PABBO BB/
PULPROG zg30
TD 65536
SOLVENT CDCl3
NS 16
DS 2
SWH 8012.820 Hz
FIDRES 0.122266 Hz
AQ 4.0894465 sec
RG 133.17
DW 62.400 usec
DE 6.50 usec
TE 297.7 K
D1 1.00000000 sec
TD0 1
======== CHANNEL f1 ========
SFO1 400.2124715 MHz
NUC1 1H
P1 15.54 usec
PLW1 12.00000000 W
F2 - Processing parameters
SI 65536
SF 400.2100000 MHz
WDW EM
SSB 0
LB 0.30 Hz
GB 0
PC 1.00
5c
10
9
8
7
6
5
4
3
2
1
ppm
1.00
1.03
2.04
4.07
3.05
2.01
6.03
3.04
7.03
4.01

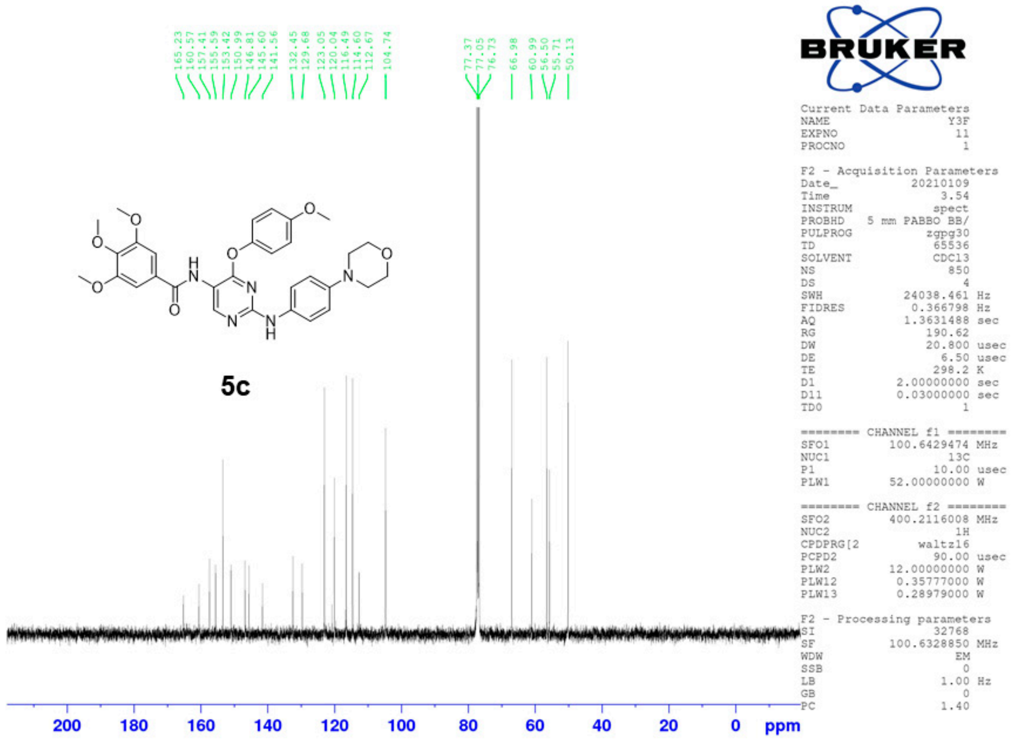
165.23
160.57
157.41
155.59
153.42
150.99
146.81
145.60
141.56
132.45
129.68
123.05
120.04
116.49
114.60
112.67
104.74
77.37
77.05
76.73
66.98
60.99
56.50
55.71
50.13
BRUKER
Current Data Parameters
NAME Y3F
EXPNO 11
PROCNO 1
F2 - Acquisition Parameters
Date_ 20210109
Time 3.54
INSTRUM spect
PROBHD 5 mm PABBO BB/
PULPROG zgpg30
TD 65536
SOLVENT CDCl3
NS 850
DS 4
SWH 24038.461 Hz
FIDRES 0.366798 Hz
AQ 1.3631488 sec
RG 190.62
DW 20.800 usec
DE 6.50 usec
TE 298.2 K
D1 2.00000000 sec
D11 0.03000000 sec
TD0 1
======== CHANNEL f1 ========
SFO1 100.6429474 MHz
NUC1 13C
P1 10.00 usec
PLW1 52.00000000 W
======== CHANNEL f2 ========
SFO2 400.2116008 MHz
NUC2 1H
CPDPRG[2 waltz16
PCPD2 90.00 usec
PLW2 12.00000000 W
PLW12 0.35777000 W
PLW13 0.28979000 W
F2 - Processing parameters
SI 32768
SF 100.6328850 MHz
WDW EM
SSB 0
LB 1.00 Hz
GB 0
PC 1.40
5c
200
180
160
140
120
100
80
60
40
20
0
ppm

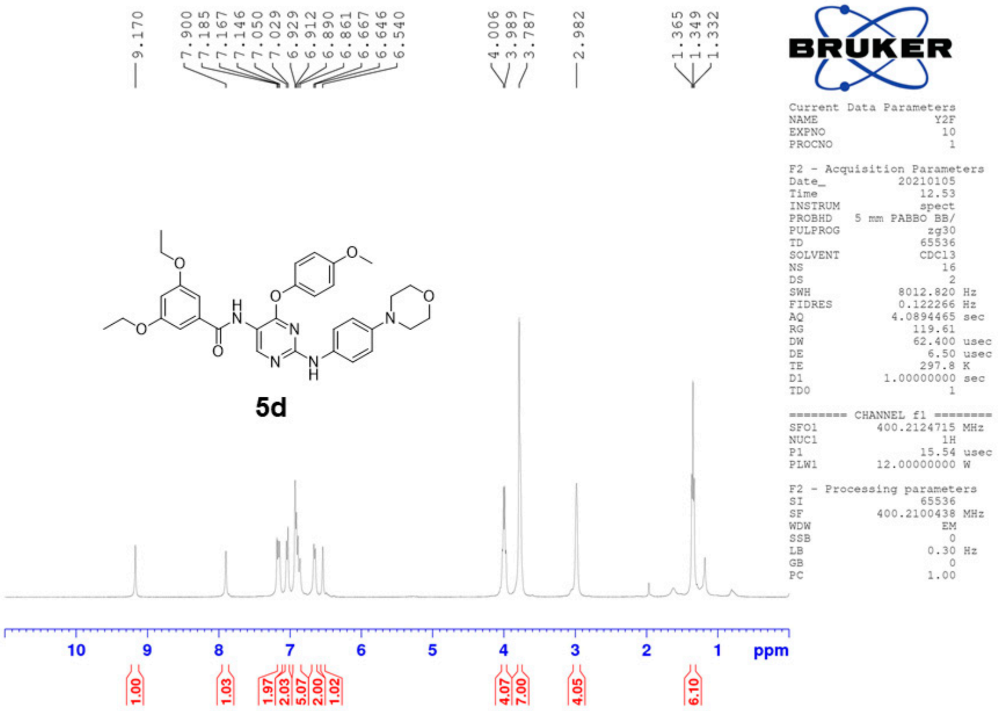
9.170
7.900
7.185
7.167
7.146
7.050
7.029
6.929
6.912
6.890
6.861
6.667
6.646
6.540
4.006
3.989
3.787
2.982
1.365
1.349
1.332
5d
10 9 8 7 6 5 4 3 2 1 ppm
1.00
1.03
1.97
2.03
5.07
2.00
1.02
4.07
7.00
4.05
6.10
BRUKER
Current Data Parameters
NAME Y2F
EXPNO 10
PROCNO 1
F2 - Acquisition Parameters
Date_ 20210105
Time 12.53
INSTRUM spect
PROBHD 5 mm PABBO BB/
PULPROG zg30
TD 65536
SOLVENT CDCl3
NS 16
DS 2
SWH 8012.820 Hz
FIDRES 0.122266 Hz
AQ 4.0894465 sec
RG 119.61
DW 62.400 usec
DE 6.50 usec
TE 297.8 K
D1 1.00000000 sec
TD0 1
======== CHANNEL f1 ========
SFO1 400.2124715 MHz
NUC1 1H
P1 15.54 usec
PLW1 12.00000000 W
F2 - Processing parameters
SI 65536
SF 400.2100438 MHz
WDW EM
SSB 0
LB 0.30 Hz
GB 0
PC 1.00

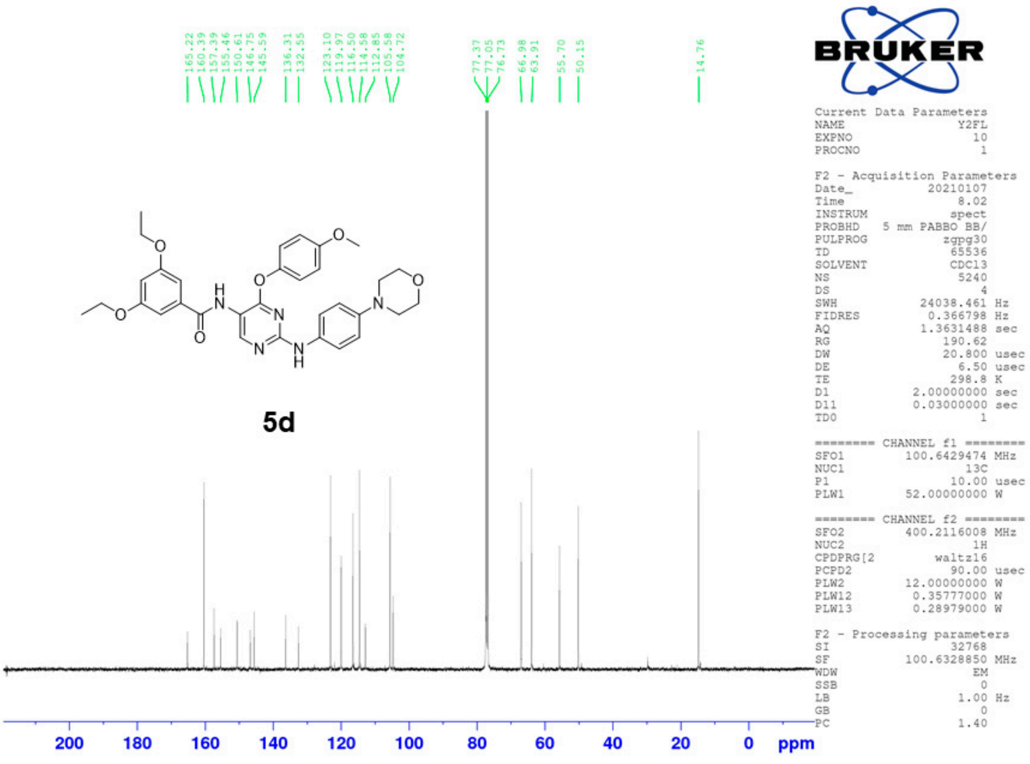
165.22
160.39
157.39
155.46
150.61
146.75
145.59
136.31
132.55
123.10
119.97
116.50
114.58
112.85
105.58
104.72
77.37
77.05
76.73
66.98
63.91
55.70
50.15
14.76
5d
200 180 160 140 120 100 80 60 40 20 0 ppm
BRUKER
Current Data Parameters
NAME Y2FL
EXPNO 10
PROCNO 1
F2 - Acquisition Parameters
Date_ 20210107
Time 8.02
INSTRUM spect
PROBHD 5 mm PABBO BB/
PULPROG zgpg30
TD 65536
SOLVENT CDCl3
NS 5240
DS 4
SWH 24038.461 Hz
FIDRES 0.366798 Hz
AQ 1.3631488 sec
RG 190.62
DW 20.800 usec
DE 6.50 usec
TE 298.8 K
D1 2.00000000 sec
D11 0.03000000 sec
TD0 1
======== CHANNEL f1 ========
SFO1 100.6429474 MHz
NUC1 13C
P1 10.00 usec
PLW1 52.00000000 W
======== CHANNEL f2 ========
SFO2 400.2116008 MHz
NUC2 1H
CPDPRG[2 waltz16
PCPD2 90.00 usec
PLW2 12.00000000 W
PLW12 0.35777000 W
PLW13 0.28979000 W
F2 - Processing parameters
SI 32768
SF 100.6328850 MHz
WDW EM
SSB 0
LB 1.00 Hz
GB 0
PC 1.40

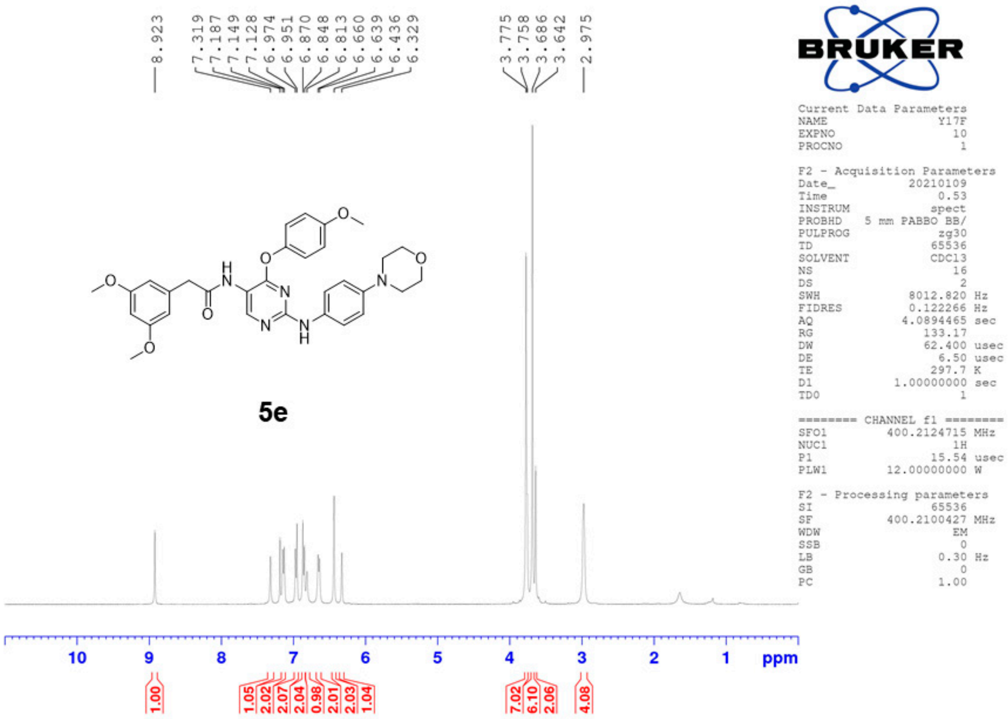
8.923
7.319 7.187 7.149 7.128 6.974 6.951 6.870 6.848 6.813 6.660 6.639 6.436 6.329
3.775 3.758 3.686 3.642
2.975
BRUKER
Current Data Parameters
NAME Y17F
EXPNO 10
PROCNO 1
F2 - Acquisition Parameters
Date_ 20210109
Time 0.53
INSTRUM spect
PROBHD 5 mm PABBO BB/
PULPROG zg30
TD 65536
SOLVENT CDCl3
NS 16
DS 2
SWH 8012.820 Hz
FIDRES 0.122266 Hz
AQ 4.0894465 sec
RG 133.17
DW 62.400 usec
DE 6.50 usec
TE 297.7 K
D1 1.00000000 sec
TD0 1
======== CHANNEL f1 ========
SFO1 400.2124715 MHz
NUC1 1H
P1 15.54 usec
PLW1 12.00000000 W
F2 - Processing parameters
SI 65536
SF 400.2100427 MHz
WDW EM
SSB 0
LB 0.30 Hz
GB 0
PC 1.00
5e
10 9 8 7 6 5 4 3 2 1 ppm
1.00
1.05 2.02 2.07 2.04 0.98 2.01 2.03 1.04
7.02 6.10 2.06
4.08

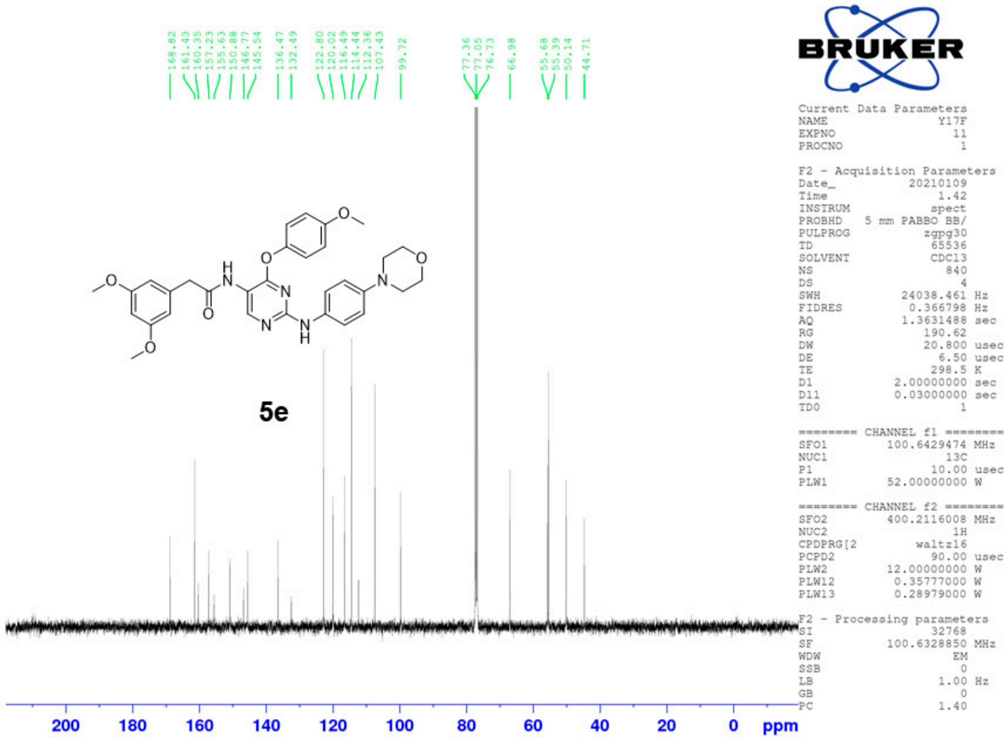
168.82 161.43 160.35 157.23 155.63 150.88 146.77 145.54
136.47 132.49
122.80 120.02 116.49 114.44 112.36 107.43
99.72
77.36 77.05 76.73
66.98
55.68 55.39 50.14
44.71
BRUKER
Current Data Parameters
NAME Y17F
EXPNO 11
PROCNO 1
F2 - Acquisition Parameters
Date_ 20210109
Time 1.42
INSTRUM spect
PROBHD 5 mm PABBO BB/
PULPROG zgpg30
TD 65536
SOLVENT CDCl3
NS 840
DS 4
SWH 24038.461 Hz
FIDRES 0.366798 Hz
AQ 1.3631488 sec
RG 190.62
DW 20.800 usec
DE 6.50 usec
TE 298.5 K
D1 2.00000000 sec
D11 0.03000000 sec
TD0 1
======== CHANNEL f1 ========
SFO1 100.6429474 MHz
NUC1 13C
P1 10.00 usec
PLW1 52.00000000 W
======== CHANNEL f2 ========
SFO2 400.2116008 MHz
NUC2 1H
CPDPRG[2 waltz16
PCPD2 90.00 usec
PLW2 12.00000000 W
PLW12 0.35777000 W
PLW13 0.28979000 W
F2 - Processing parameters
SI 32768
SF 100.6328850 MHz
WDW EM
SSB 0
LB 1.00 Hz
GB 0
PC 1.40
5e
200 180 160 140 120 100 80 60 40 20 0 ppm

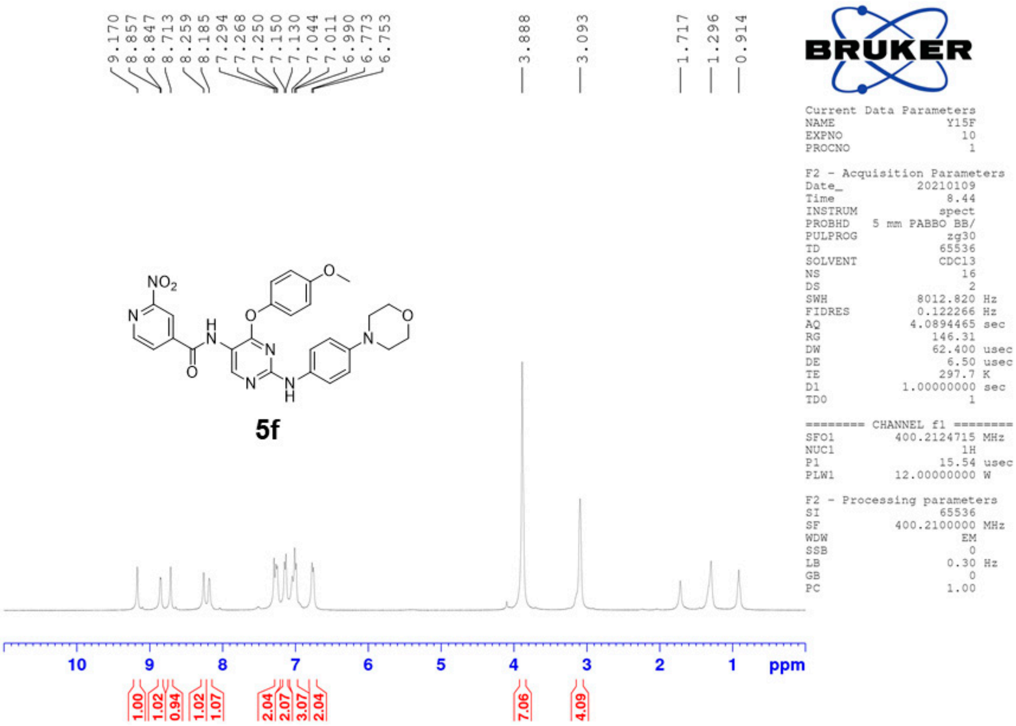
9.170
8.857
8.847
8.713
8.259
8.185
7.294
7.268
7.250
7.150
7.130
7.044
7.011
6.990
6.773
6.753
3.888
3.093
1.717
1.296
0.914
BRUKER
Current Data Parameters
NAME Y15F
EXPNO 10
PROCNO 1
F2 - Acquisition Parameters
Date_ 20210109
Time 8.44
INSTRUM spect
PROBHD 5 mm PABBO BB/
PULPROG zg30
TD 65536
SOLVENT CDCl3
NS 16
DS 2
SWH 8012.820 Hz
FIDRES 0.122266 Hz
AQ 4.0894465 sec
RG 146.31
DW 62.400 usec
DE 6.50 usec
TE 297.7 K
D1 1.00000000 sec
TD0 1
======== CHANNEL f1 ========
SFO1 400.2124715 MHz
NUC1 1H
P1 15.54 usec
PLW1 12.00000000 W
F2 - Processing parameters
SI 65536
SF 400.2100000 MHz
WDW EM
SSB 0
LB 0.30 Hz
GB 0
PC 1.00
NO2
N
H
N
O
O
O
N
N
N
H
N
O
5f
10
9
8
7
6
5
4
3
2
1
ppm
1.00
1.02
0.94
1.02
1.07
2.04
2.07
3.07
2.04
7.06
4.09

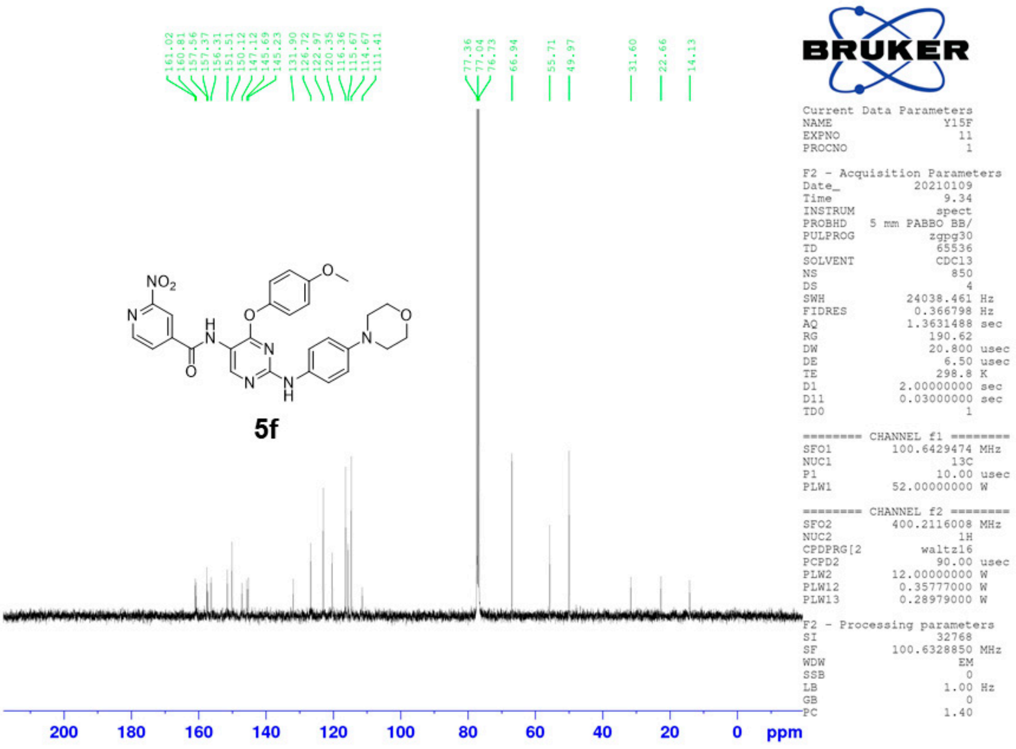
161.02
160.81
157.56
157.37
156.31
151.51
150.12
147.12
145.69
145.23
131.90
126.72
122.97
120.35
116.36
115.67
114.67
111.41
77.36
77.04
76.73
66.94
55.71
49.97
31.60
22.66
14.13
BRUKER
Current Data Parameters
NAME Y15F
EXPNO 11
PROCNO 1
F2 - Acquisition Parameters
Date_ 20210109
Time 9.34
INSTRUM spect
PROBHD 5 mm PABBO BB/
PULPROG zgpg30
TD 65536
SOLVENT CDCl3
NS 850
DS 4
SWH 24038.461 Hz
FIDRES 0.366798 Hz
AQ 1.3631488 sec
RG 190.62
DW 20.800 usec
DE 6.50 usec
TE 298.8 K
D1 2.00000000 sec
D11 0.03000000 sec
TD0 1
======== CHANNEL f1 ========
SFO1 100.6429474 MHz
NUC1 13C
P1 10.00 usec
PLW1 52.00000000 W
======== CHANNEL f2 ========
SFO2 400.2116008 MHz
NUC2 1H
CPDPRG[2 waltz16
PCPD2 90.00 usec
PLW2 12.00000000 W
PLW12 0.35777000 W
PLW13 0.28979000 W
F2 - Processing parameters
SI 32768
SF 100.6328850 MHz
WDW EM
SSB 0
LB 1.00 Hz
GB 0
PC 1.40
NO2
N
H
N
O
O
O
N
N
N
H
N
O
5f
200
180
160
140
120
100
80
60
40
20
0
ppm

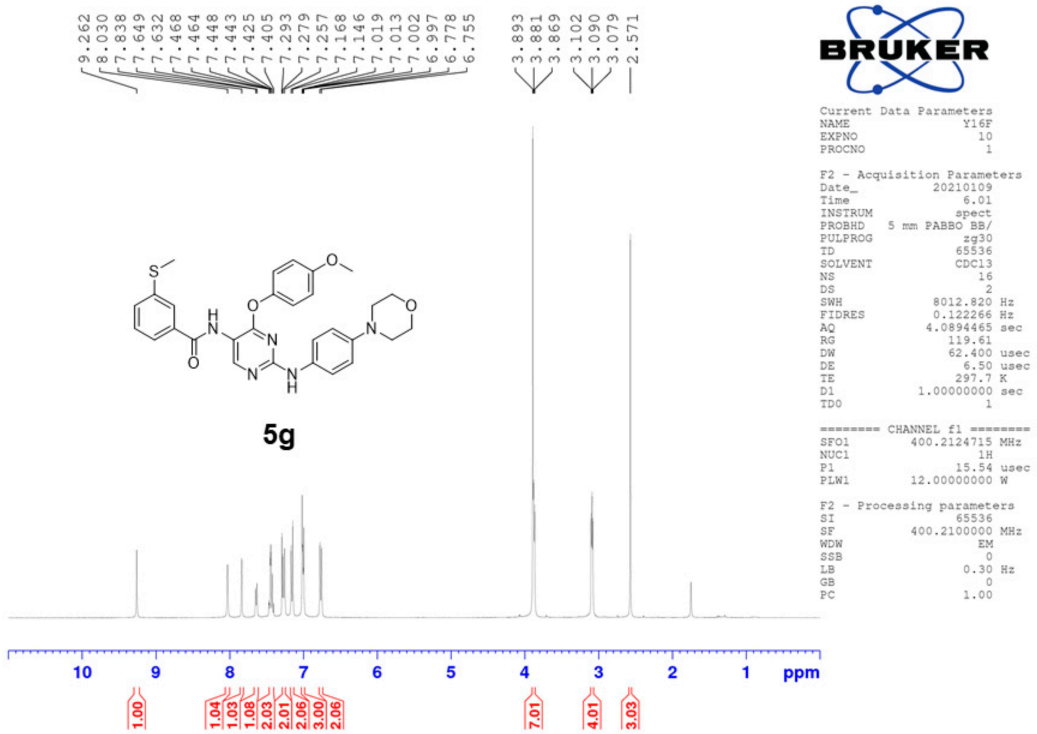
5g
Current Data Parameters
NAME Y16F
EXPNO 10
PROCNO 1
F2 - Acquisition Parameters
Date_ 20210109
Time 6.01
INSTRUM spect
PROBHD 5 mm PABBO BB/
PULPROG zg30
TD 65536
SOLVENT CDCl3
NS 16
DS 2
SWH 8012.820 Hz
FIDRES 0.122266 Hz
AQ 4.0894465 sec
RG 119.61
DW 62.400 usec
DE 6.50 usec
TE 297.7 K
D1 1.00000000 sec
TD0 1
CHANNEL f1
SFO1 400.2124715 MHz
NUC1 1H
P1 15.54 usec
PLW1 12.00000000 W
F2 - Processing parameters
SI 65536
SF 400.2100000 MHz
WDW EM
SSB 0
LB 0.30 Hz
GB 0
PC 1.00
ppm

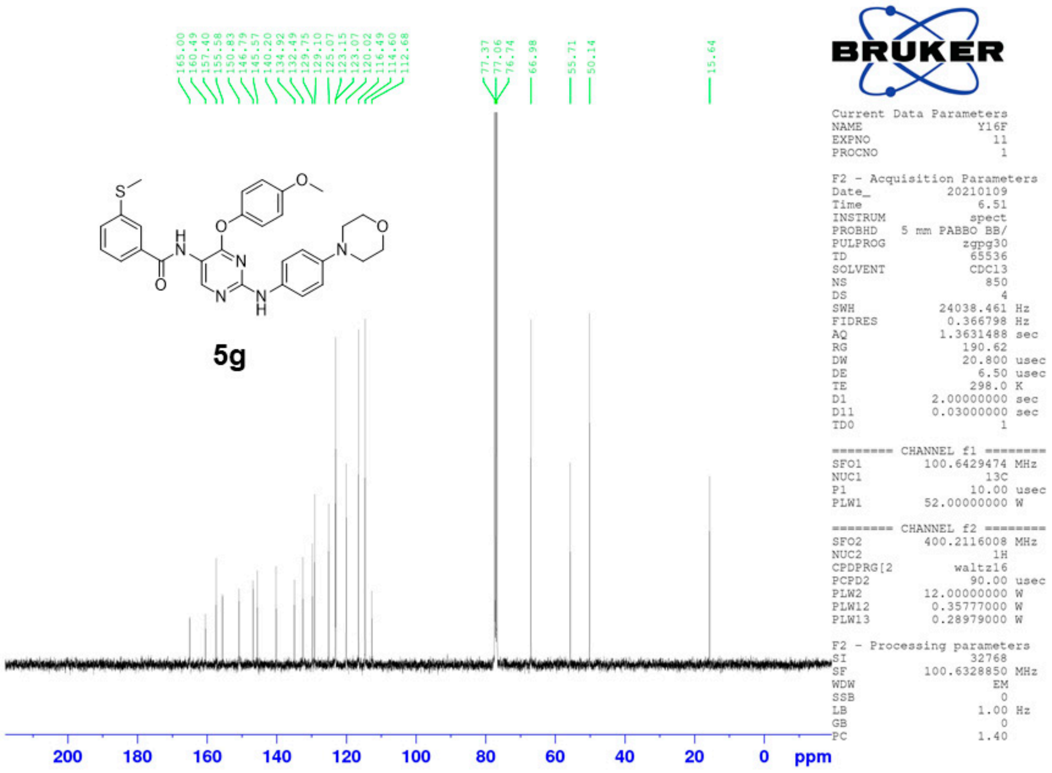
5g
Current Data Parameters
NAME Y16F
EXPNO 11
PROCNO 1
F2 - Acquisition Parameters
Date_ 20210109
Time 6.51
INSTRUM spect
PROBHD 5 mm PABBO BB/
PULPROG zgpg30
TD 65536
SOLVENT CDCl3
NS 850
DS 4
SWH 24038.461 Hz
FIDRES 0.366798 Hz
AQ 1.3631488 sec
RG 190.62
DW 20.800 usec
DE 6.50 usec
TE 298.0 K
D1 2.00000000 sec
D11 0.03000000 sec
TD0 1
CHANNEL f1
SFO1 100.6429474 MHz
NUC1 13C
P1 10.00 usec
PLW1 52.00000000 W
CHANNEL f2
SFO2 400.2116008 MHz
NUC2 1H
CPDPRG[2 waltz16
PCPD2 90.00 usec
PLW2 12.00000000 W
PLW12 0.35777000 W
PLW13 0.28979000 W
F2 - Processing parameters
SI 32768
SF 100.6328850 MHz
WDW EM
SSB 0
LB 1.00 Hz
GB 0
PC 1.40
ppm

3. LC and MS conditions

LC Condition (Waters® ACQUITY™ UPLC)			
Column	CORTECS® UPLC® C18 1.6 µm (2.1x100 mm)		
	Temperature : 40 °C		
Mobile phase	A : 0.1% formic acid in water, v/v		
	B : 0.1% formic acid in methanol, v/v		
Gradient	Time (min)	A (%)	B (%)
	0.0	99.0	1.0
	1.0	99.0	1.0
	7.0	1.0	99.0
	8.5	1.0	99.0
	9.0	99.0	1.0
	10.0	99.0	1.0
Flow rate	0.3 ml/min		
Injection volume	5 µl		
**MS Condition (SYNAPT™ G2)**			
Ionization Mode	ESI+		
Capillary	+3.1 kv		
Sampling cone	40 V		
Extraction cone	4.0		
Source	120 °C		
Desolvation	350 °C		
Cone gas	100 L/h		
Desolvation gas	800 L/h		

Chromatogram_blank

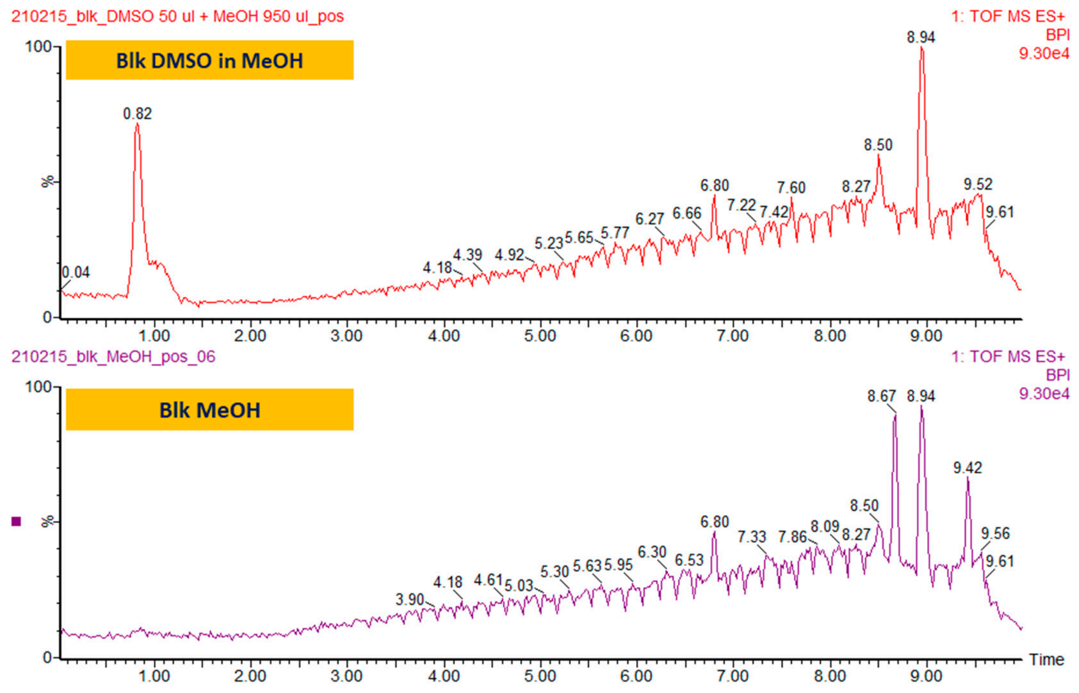

4. Representative purity and HRMS data of compounds 5a–g

Peak area(%)_ Y14

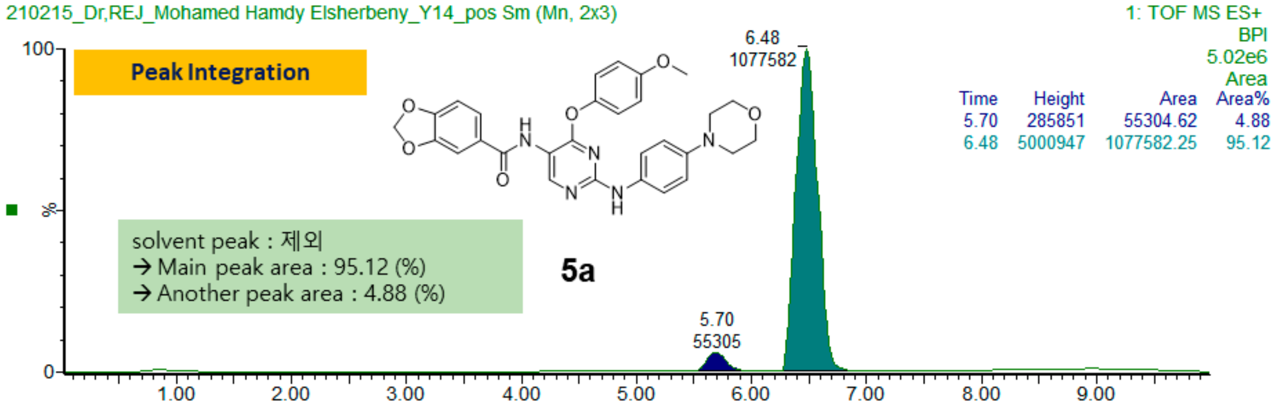

MS Spectrum_ Isotope modeling_ Y14

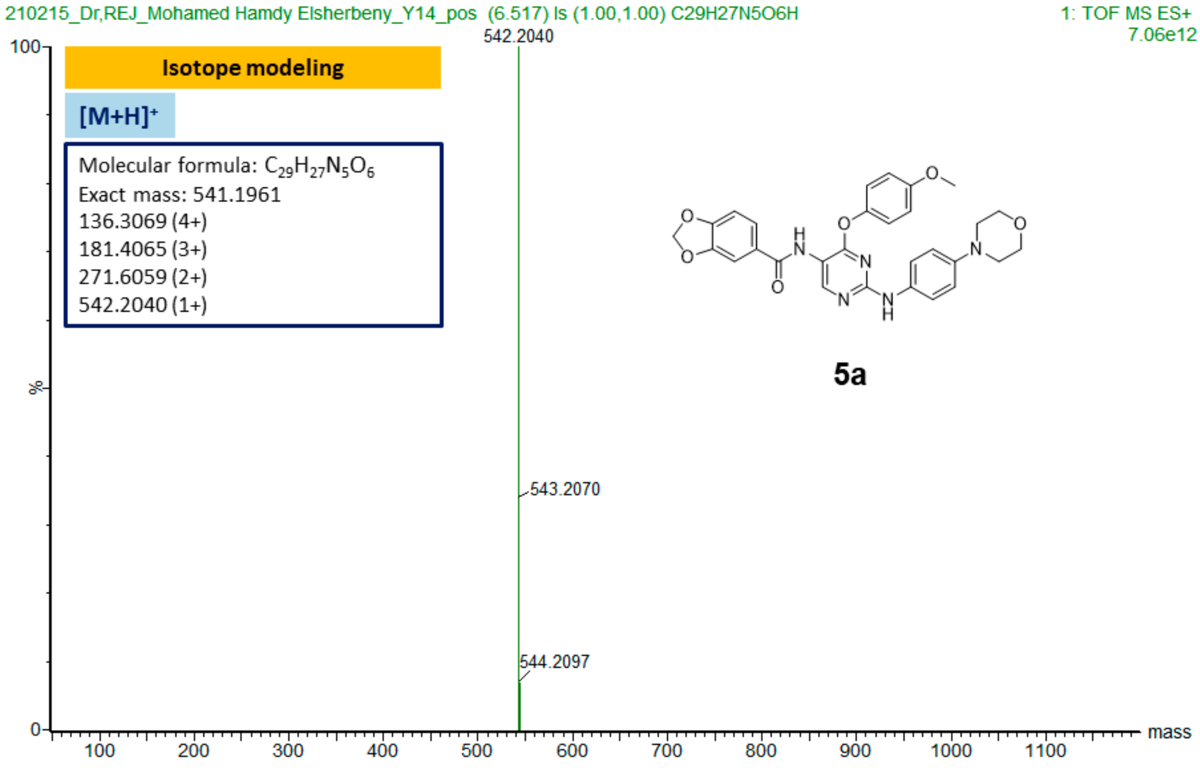

Peak area(%)_ Y5

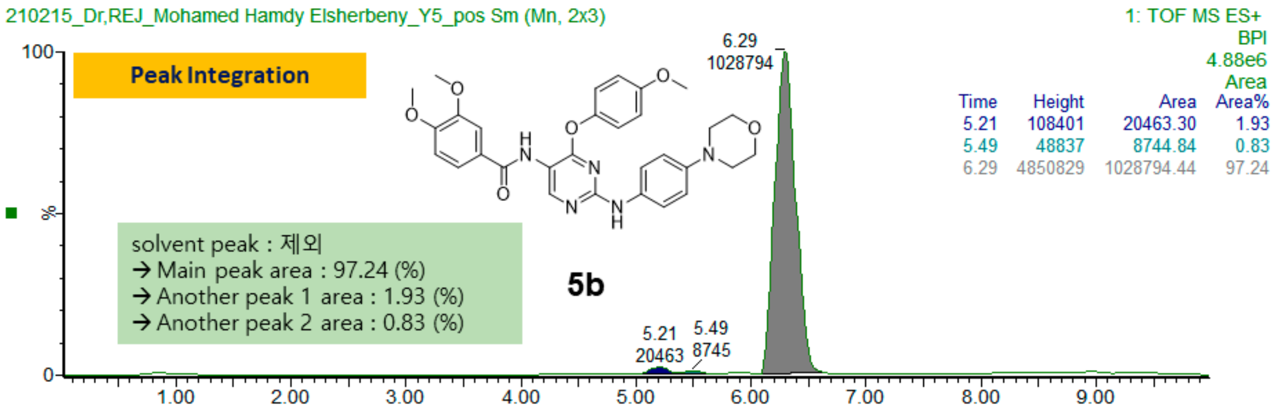

MS Spectrum_ Isotope modeling_ Y5

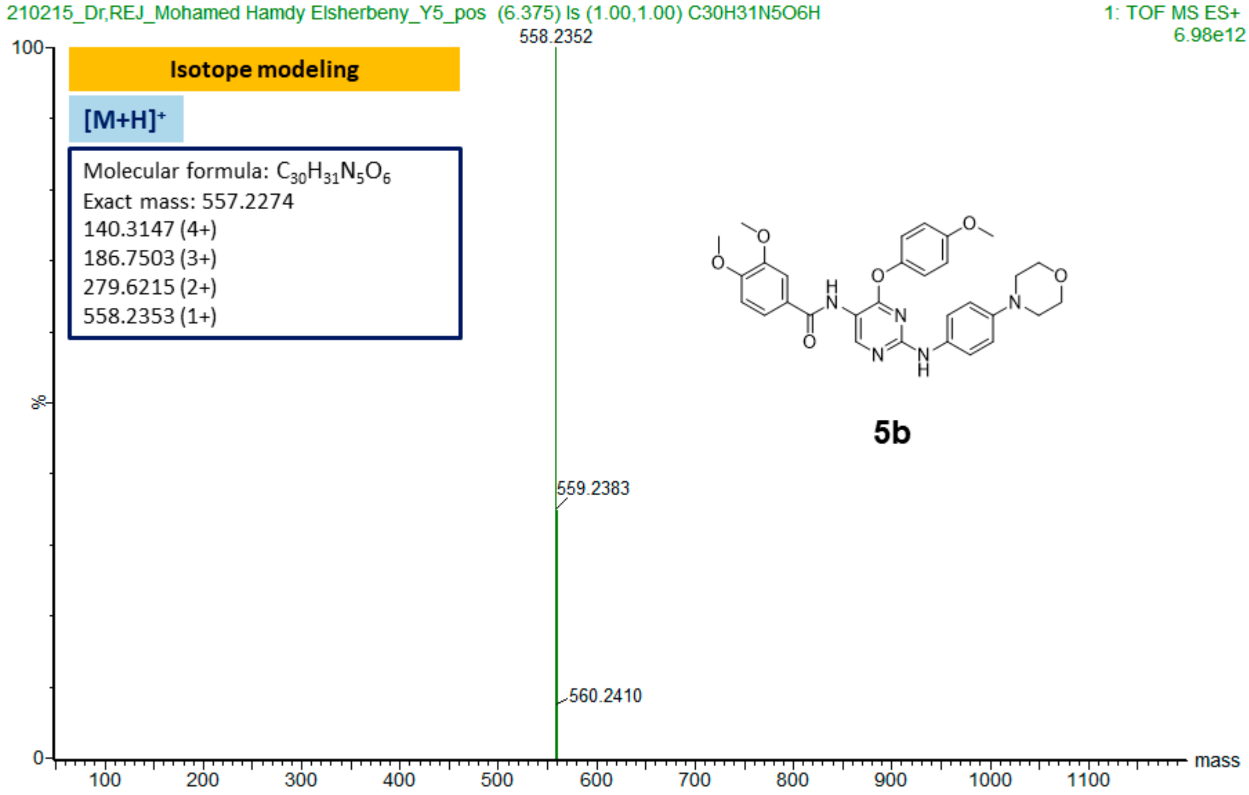

MS Spectrum_ Isotope modeling_ Y3

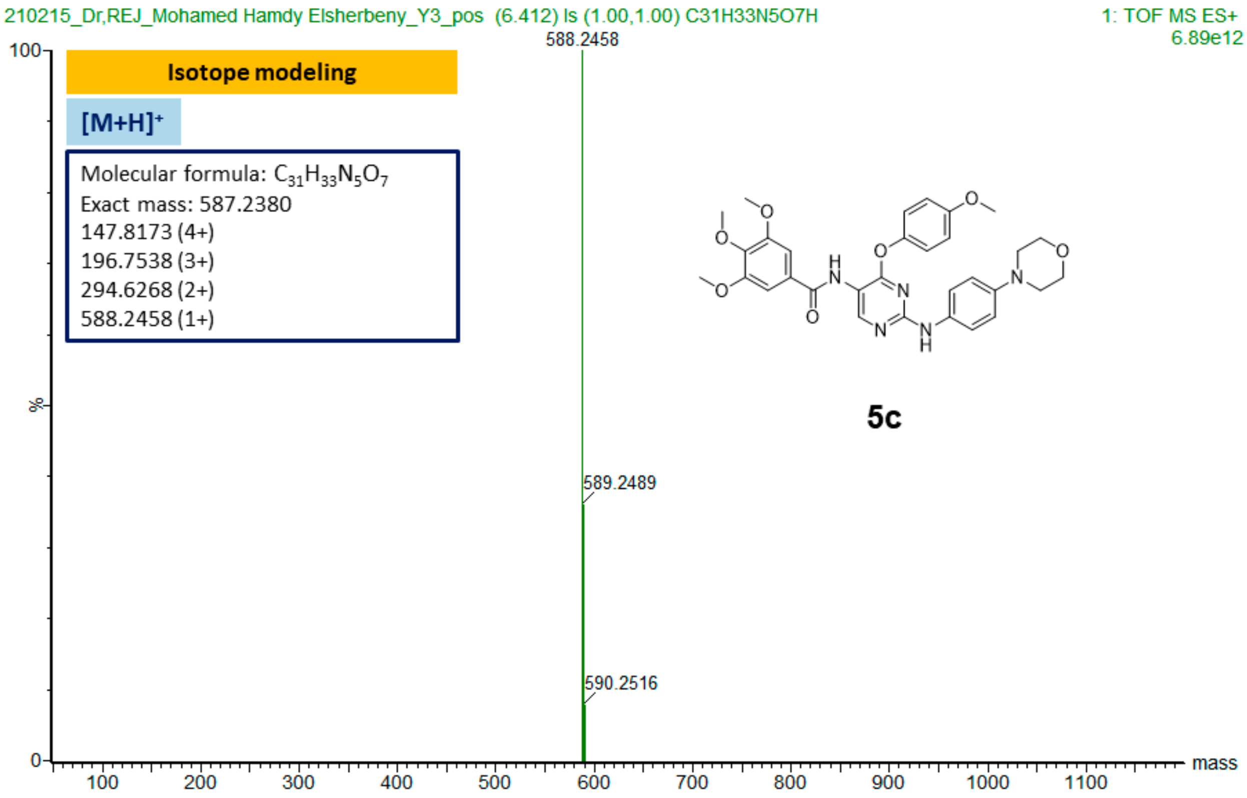

MS Spectrum_ Isotope modeling_ Y2

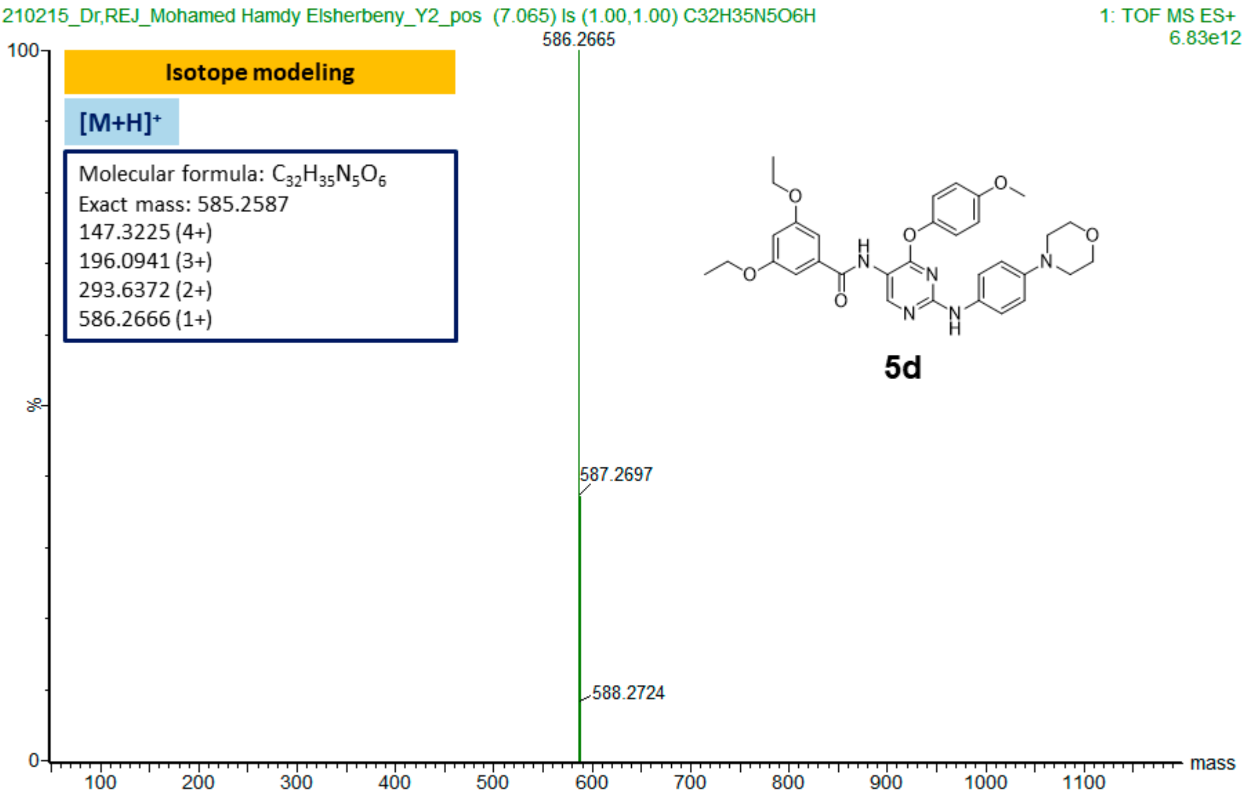

5d

Peak area(%)_ Y17

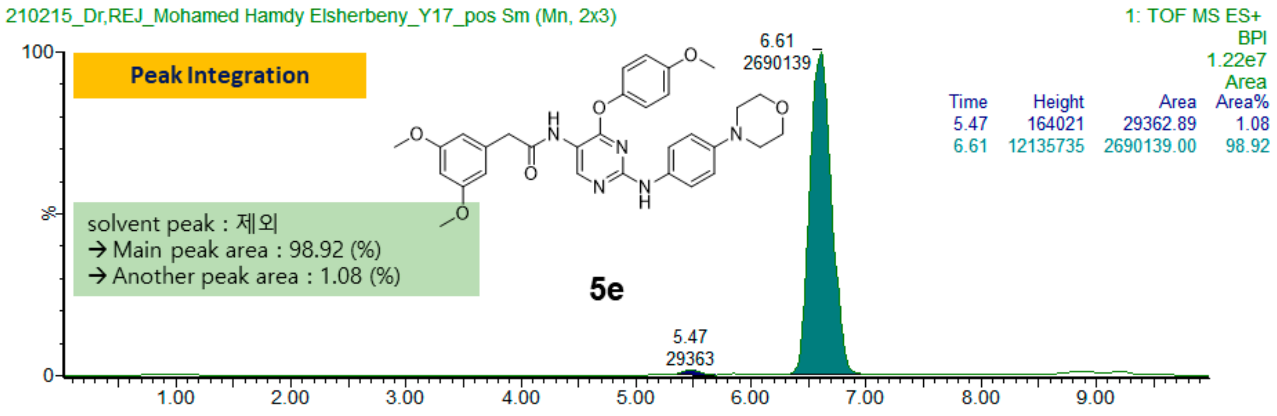

MS Spectrum_ Isotope modeling_ Y17

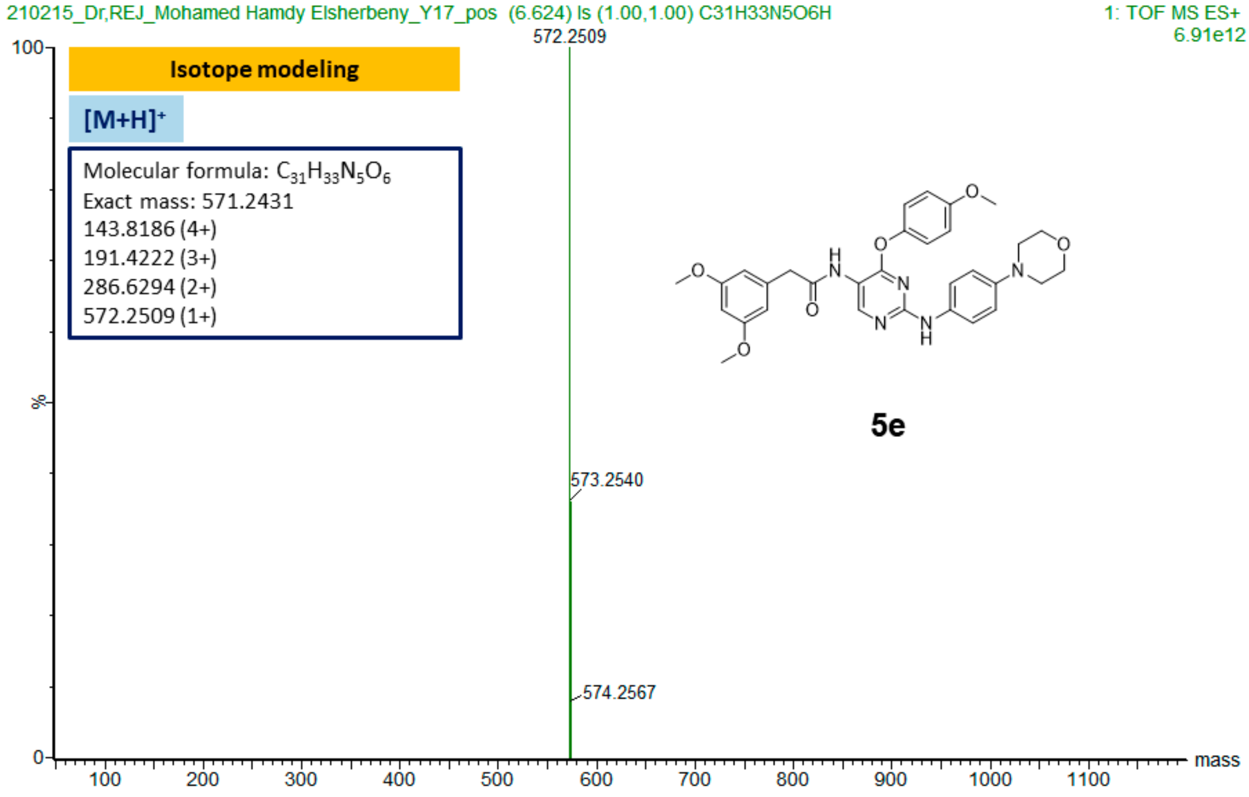

MS Spectrum_ Isotope modeling_ Y15

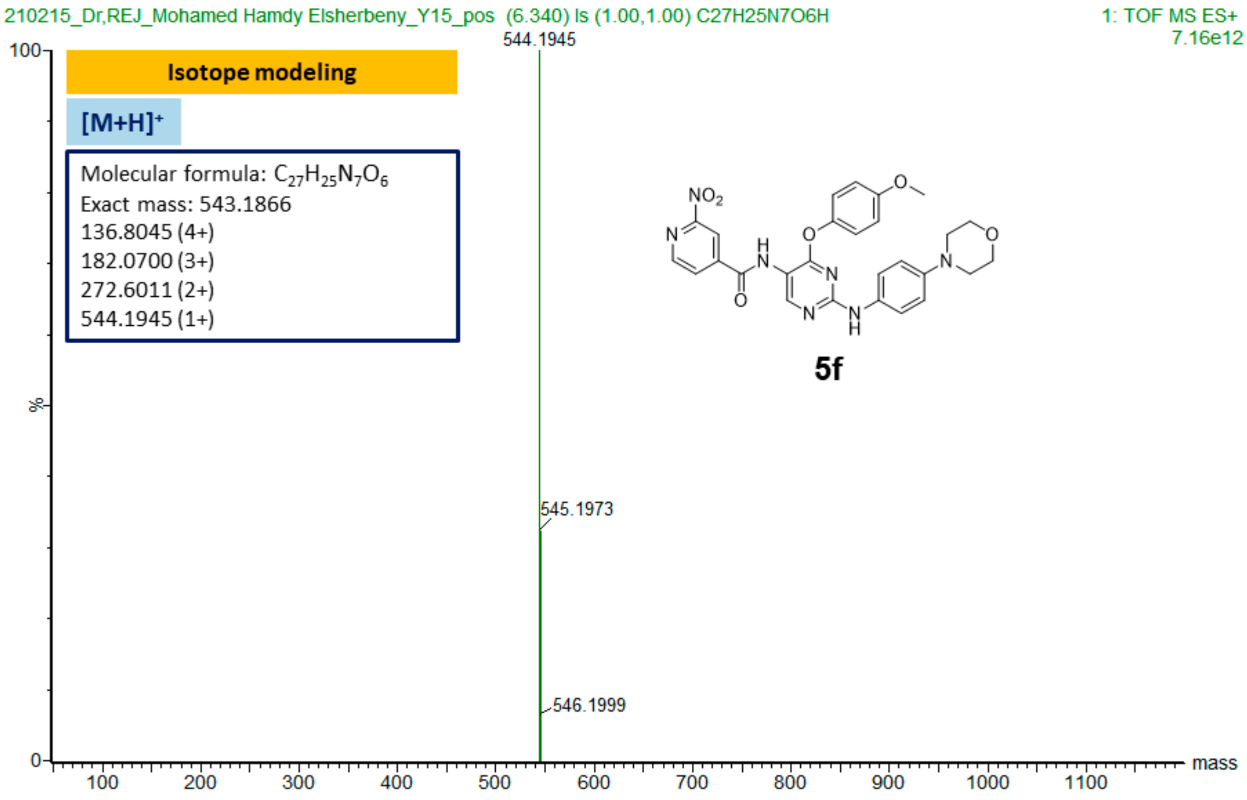

Peak area(%)_ Y16

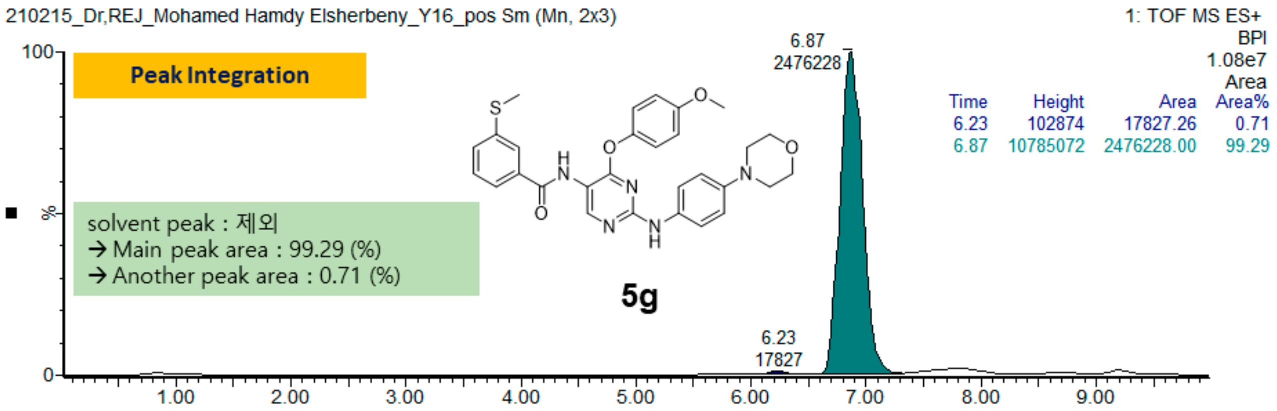

MS Spectrum_ Isotope modeling_ Y16

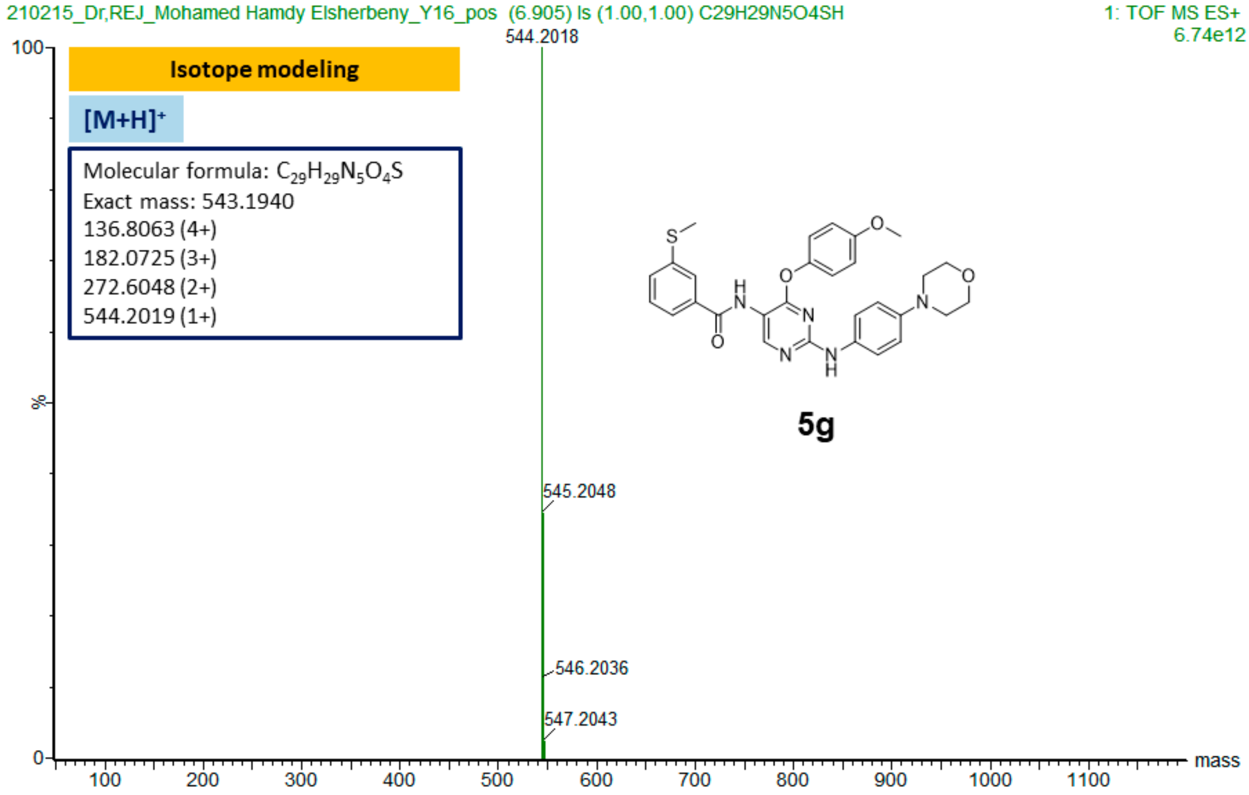

5. Protocol of Kinase assay

Kinase Profiling is 10 dose IC_{50} singlet assay. Kinases inhibitory activities were assessed by the HotSpot assay platform, which contained specific kinase/substrate pairs along with required cofactors. Base reaction buffer: 20 mM Hepes (pH 7.5), 10 mM $MgCl_2$, 1 mM EGTA, 0.02% Brij35, 0.02 mg/ml BSA, 0.1 mM Na_3VO_4, 2 mM DTT, 1% DMSO. Testing compounds were dissolved in 100% DMSO to specific concentration. The serial dilution was conducted by Integra Viaflo Assist in DMSO. The reaction mixture containing the examined compound and 33P-ATP was incubated at room temperature for 2h and radioactivity was detected by filter-binding method. Kinase activity data were expressed as the percent remaining kinase activity in test samples compared to vehicle (dimethyl sulfoxide) reactions. IC_{50} values and curve fits were obtained using Prism (GraphPad Software).

6. Biology raw data for IC_{50} determination

Paper ID	Cpd ID	NSC Number
**5d**	DK_Y2	827664
**5c**	DK_Y3	827665
**5b**	DK_Y5	827666
**5a**	DK_Y14	827667
**5f**	DK_Y15	827668
**5g**	DK_Y16	827669
**5e**	DK_Y17	827670

	A	B	C	D	E	F
1	**Kinase Profiling Report for:**			**LYN**		
2						
3	**Raw data**					
4		**Conc.(M)**	**DK_Y16**	**DK_Y16**	**Staurosporine**	**Stauro Conc.(M)**
5		**1.00E-04**	102058	97795	457	**2.00E-05**
6		**3.33E-05**	91411	96509	4781	**5.00E-06**
7		**1.11E-05**	99636	88012	2031	**1.25E-06**
8		**3.70E-06**	107790	123234	5061	**3.13E-07**
9		**1.23E-06**	402276	397973	17915	**7.81E-08**
10		**4.12E-07**	1012948	1008099	85789	**1.95E-08**
11		**1.37E-07**	1424580	1521179	415780	**4.88E-09**
12		**4.57E-08**	2039036	2069042	1312529	**1.22E-09**
13		**1.52E-08**	2645282	2501772	2138576	**3.05E-10**
14		**5.08E-09**	2797609	2601868	2360674	**7.63E-11**
15		**DMSO**	2948480	2640293	2536190	**DMSO**
16						
17						
18	**%Activity**					
19		**Conc.(M)**	**DK_Y16**	**DK_Y16**	**Staurosporine**	**Stauro Conc.(M)**
20		**1.00E-04**	3.77	3.61	0.02	**2.00E-05**
21		**3.33E-05**	3.38	3.56	0.18	**5.00E-06**
22		**1.11E-05**	3.68	3.25	0.08	**1.25E-06**
23		**3.70E-06**	3.98	4.55	0.19	**3.13E-07**
24		**1.23E-06**	14.85	14.69	0.66	**7.81E-08**
25		**4.12E-07**	37.40	37.22	3.17	**1.95E-08**
26		**1.37E-07**	52.60	56.17	15.35	**4.88E-09**
27		**4.57E-08**	75.29	76.40	48.46	**1.22E-09**
28		**1.52E-08**	97.67	92.37	78.96	**3.05E-10**
29		**5.08E-09**	103.30	96.07	87.16	**7.63E-11**
30		**DMSO**	108.87	97.49	93.64	**DMSO**
31						
32		**HILLSLOPE**	**-0.80**	**-0.90**	**-1.20**	
33		**IC50 (M)**	**1.38E-07**	**2.00E-07**	**1.32E-09**	
34						
35						

Summary LCK **LYN**

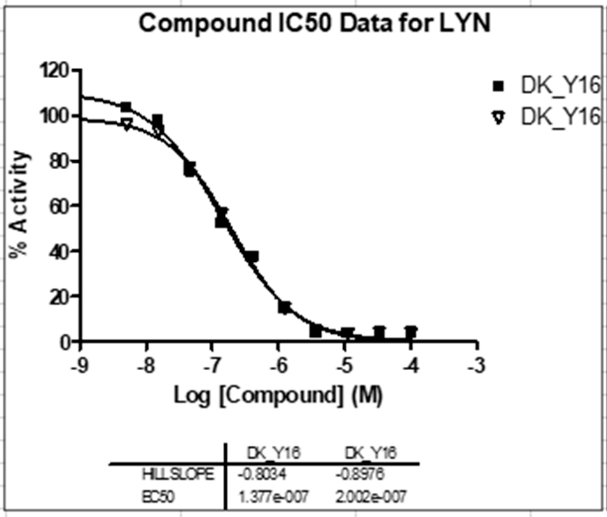

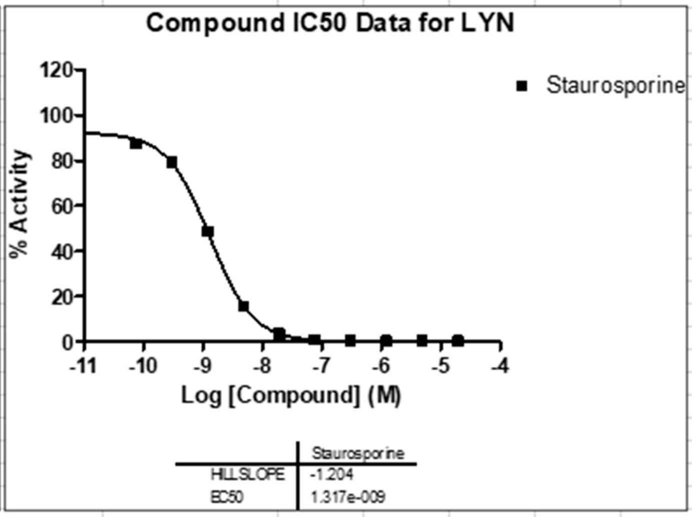

	A	B	C	D	E	F
1	**Kinase Profiling Report for:**			**LCK**		
2						
3	**Raw data**					
4		**Conc.(M)**	**DK_Y16**	**DK_Y16**	**Staurosporine**	**Stauro Conc.(M)**
5		**1.00E-04**	23600	28153	2798	**2.00E-05**
6		**3.33E-05**	25579	22567	2536	**5.00E-06**
7		**1.11E-05**	24308	24444	3537	**1.25E-06**
8		**3.70E-06**	24200	25454	6391	**3.13E-07**
9		**1.23E-06**	87291	97081	16456	**7.81E-08**
10		**4.12E-07**	283755	269570	62683	**1.95E-08**
11		**1.37E-07**	625889	650022	379559	**4.88E-09**
12		**4.57E-08**	984306	991895	835742	**1.22E-09**
13		**1.52E-08**	1067683	1298284	1382806	**3.05E-10**
14		**5.08E-09**	1392250	1385928	1452961	**7.63E-11**
15		**DMSO**	1517343	1516956	1515635	**DMSO**
16						
17						
18	**%Activity**					
19		**Conc.(M)**	**DK_Y16**	**DK_Y16**	**Staurosporine**	**Stauro Conc.(M)**
20		**1.00E-04**	1.50	1.79	0.18	**2.00E-05**
21		**3.33E-05**	1.63	1.44	0.16	**5.00E-06**
22		**1.11E-05**	1.55	1.56	0.23	**1.25E-06**
23		**3.70E-06**	1.54	1.62	0.41	**3.13E-07**
24		**1.23E-06**	5.56	6.19	1.05	**7.81E-08**
25		**4.12E-07**	18.08	17.18	3.99	**1.95E-08**
26		**1.37E-07**	39.89	41.43	24.19	**4.88E-09**
27		**4.57E-08**	62.73	63.21	53.26	**1.22E-09**
28		**1.52E-08**	68.04	82.74	88.12	**3.05E-10**
29		**5.08E-09**	88.73	88.32	92.60	**7.63E-11**
30		**DMSO**	96.70	96.67	96.59	**DMSO**
31						
32		**HILLSLOPE**	**-0.83**	**-0.98**	**-1.14**	
33		**IC50 (M)**	**7.94E-08**	**9.59E-08**	**1.63E-09**	
34						
35						

Summary LCK LYN

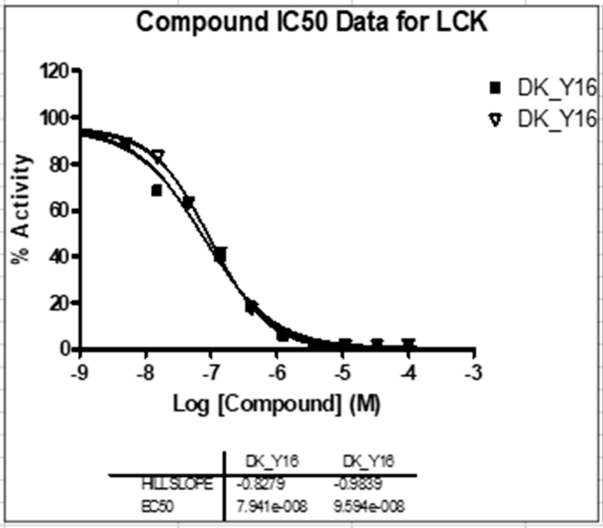

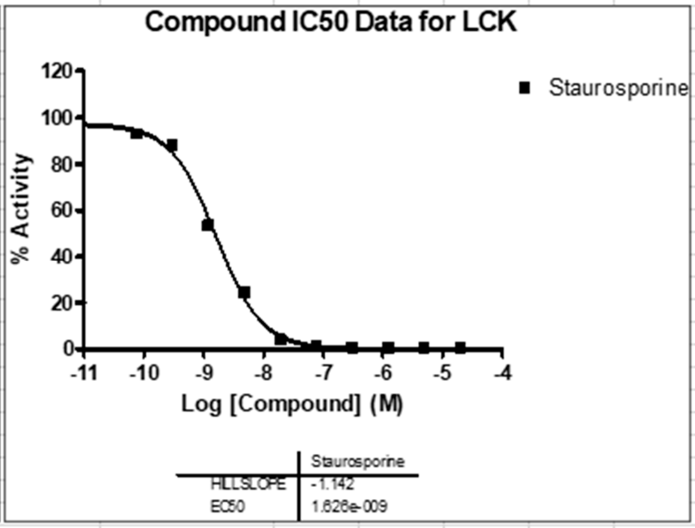

	A	B	C	D	E	F	G	H
1	**Kinase Profiling Report for:**			**FMS**				
2								
3	**Raw data**							
4		**Conc.(M)**	**DK_Y2**	**DK_Y2**	**DK_Y16**	**DK_Y16**	**Staurosporine**	**Stauro Conc.(M)**
5		**1.00E-04**	-97	-1909	9816	9452	-1707	**2.00E-05**
6		**3.33E-05**	2760	4094	12057	11554	-7936	**5.00E-06**
7		**1.11E-05**	10980	9617	7998	7906	-3557	**1.25E-06**
8		**3.70E-06**	39265	31468	15729	18380	-3529	**3.13E-07**
9		**1.23E-06**	76846	77042	49210	48785	-3941	**7.81E-08**
10		**4.12E-07**	177528	156344	119524	112724	7057	**1.95E-08**
11		**1.37E-07**	289596	324465	233088	208611	72113	**4.88E-09**
12		**4.57E-08**	395925	391901	341069	349825	289324	**1.22E-09**
13		**1.52E-08**	446517	436856	426845	397685	186	**3.05E-10**
14		**5.08E-09**	491433	487793	464322	466912	493584	**7.63E-11**
15		**DMSO**	478093	504603	491922	498472	525118	**DMSO**
16								
17								
18	**%Activity**						Value excluded from curve fit	
19		**Conc.(M)**	**DK_Y2**	**DK_Y2**	**DK_Y16**	**DK_Y16**	**Staurosporine**	**Stauro Conc.(M)**
20		**1.00E-04**	-0.02	-0.38	1.96	1.89	-0.34	**2.00E-05**
21		**3.33E-05**	0.55	0.82	2.41	2.31	-1.59	**5.00E-06**
22		**1.11E-05**	2.20	1.92	1.60	1.58	-0.71	**1.25E-06**
23		**3.70E-06**	7.86	6.30	3.15	3.68	-0.71	**3.13E-07**
24		**1.23E-06**	15.38	15.42	9.85	9.76	-0.79	**7.81E-08**
25		**4.12E-07**	35.53	31.29	23.92	22.56	1.41	**1.95E-08**
26		**1.37E-07**	57.96	64.94	46.65	41.75	14.43	**4.88E-09**
27		**4.57E-08**	79.24	78.44	68.26	70.02	57.91	**1.22E-09**
28		**1.52E-08**	89.37	87.43	85.43	79.59	0.04	**3.05E-10**
29		**5.08E-09**	98.36	97.63	92.93	93.45	98.79	**7.63E-11**
30		**DMSO**	95.69	100.99	98.45	99.77	105.10	**DMSO**
31								
32		**HILLSLOPE**	**-0.93**	**-0.95**	**-0.91**	**-0.87**	**-1.47**	
33		**IC50 (M)**	**2.14E-07**	**2.12E-07**	**1.18E-07**	**1.02E-07**	**1.44E-09**	
34								

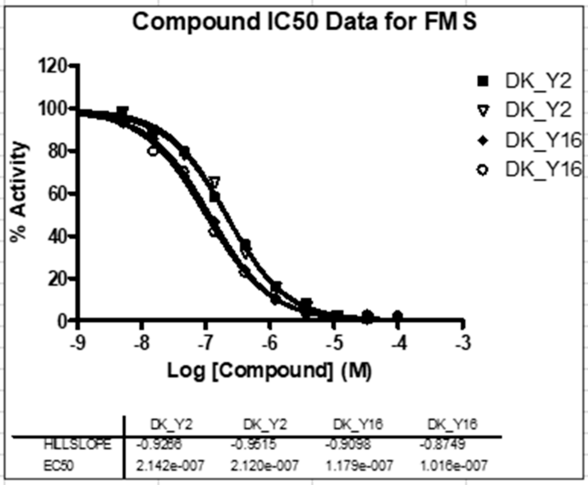

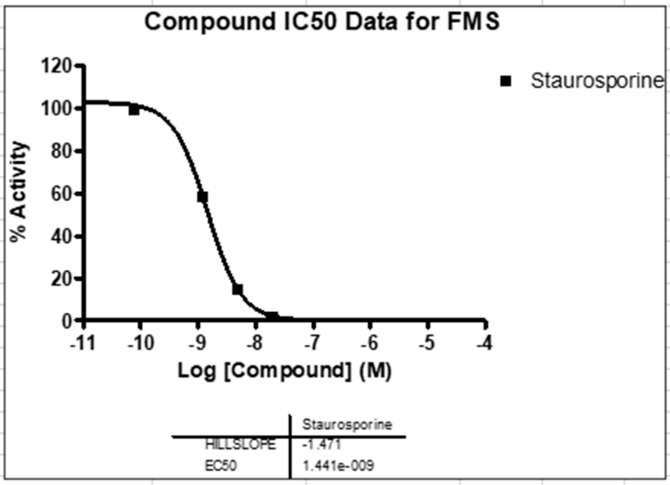

7. Antitumor data sheets carried out at NCI, USA.

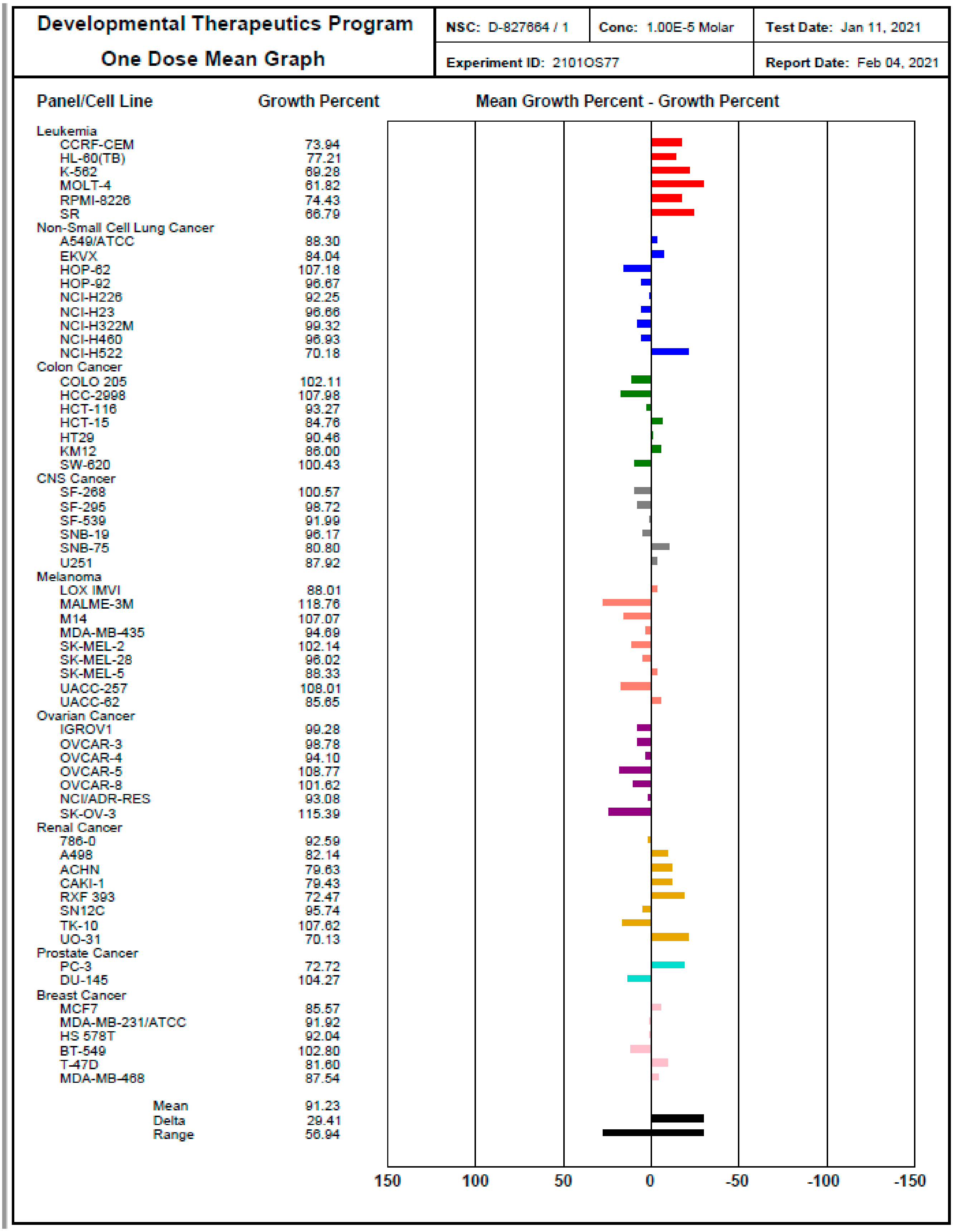
Developmental Therapeutics Program
One Dose Mean Graph
NSC: D-827664 / 1
Conc: 1.00E-5 Molar
Test Date: Jan 11, 2021
Experiment ID: 2101OS77
Report Date: Feb 04, 2021
Panel/Cell Line
Growth Percent
Mean Growth Percent - Growth Percent
Leukemia
CCRF-CEM 73.94
HL-60(TB) 77.21
K-562 69.28
MOLT-4 61.82
RPMI-8226 74.43
SR 66.79
Non-Small Cell Lung Cancer
A549/ATCC 88.30
EKVX 84.04
HOP-62 107.18
HOP-92 96.67
NCI-H226 92.25
NCI-H23 96.66
NCI-H322M 99.32
NCI-H460 96.93
NCI-H522 70.18
Colon Cancer
COLO 205 102.11
HCC-2998 107.98
HCT-116 93.27
HCT-15 84.76
HT29 90.46
KM12 86.00
SW-620 100.43
CNS Cancer
SF-268 100.57
SF-295 98.72
SF-539 91.99
SNB-19 96.17
SNB-75 80.80
U251 87.92
Melanoma
LOX IMVI 88.01
MALME-3M 118.76
M14 107.07
MDA-MB-435 94.69
SK-MEL-2 102.14
SK-MEL-28 96.02
SK-MEL-5 88.33
UACC-257 108.01
UACC-62 85.65
Ovarian Cancer
IGROV1 99.28
OVCAR-3 98.78
OVCAR-4 94.10
OVCAR-5 108.77
OVCAR-8 101.62
NCI/ADR-RES 93.08
SK-OV-3 115.39
Renal Cancer
786-0 92.59
A498 82.14
ACHN 79.63
CAKI-1 79.43
RXF 393 72.47
SN12C 95.74
TK-10 107.62
UO-31 70.13
Prostate Cancer
PC-3 72.72
DU-145 104.27
Breast Cancer
MCF7 85.57
MDA-MB-231/ATCC 91.92
HS 578T 92.04
BT-549 102.80
T-47D 81.60
MDA-MB-468 87.54
Mean 91.23
Delta 29.41
Range 56.94
150
100
50
0
-50
-100
-150

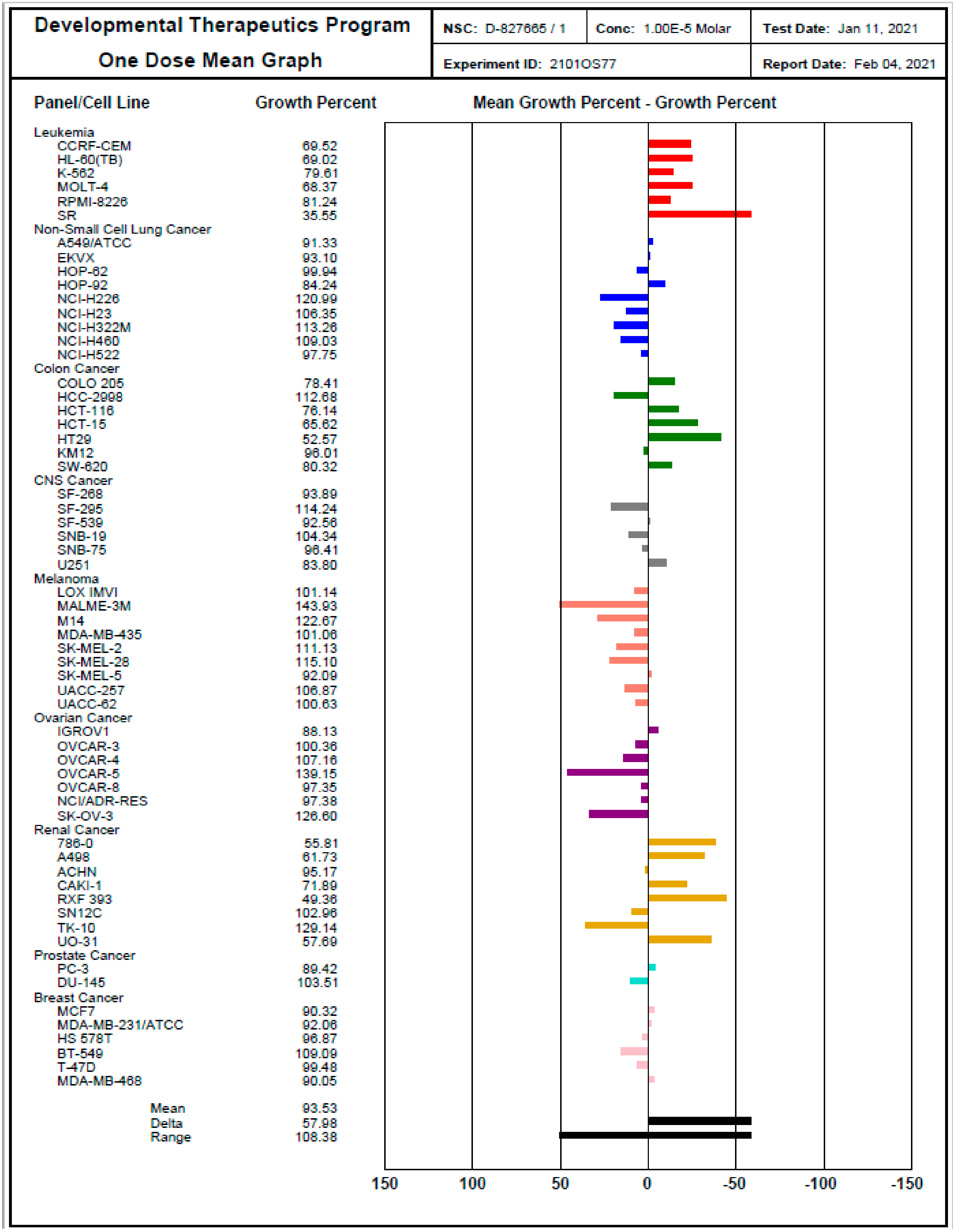
Developmental Therapeutics Program
One Dose Mean Graph
NSC: D-827665 / 1
Conc: 1.00E-5 Molar
Test Date: Jan 11, 2021
Experiment ID: 2101OS77
Report Date: Feb 04, 2021
Panel/Cell Line
Growth Percent
Mean Growth Percent - Growth Percent
Leukemia
CCRF-CEM 69.52
HL-60(TB) 69.02
K-562 79.61
MOLT-4 68.37
RPMI-8226 81.24
SR 35.55
Non-Small Cell Lung Cancer
A549/ATCC 91.33
EKVX 93.10
HOP-62 99.94
HOP-92 84.24
NCI-H226 120.99
NCI-H23 106.35
NCI-H322M 113.26
NCI-H460 109.03
NCI-H522 97.75
Colon Cancer
COLO 205 78.41
HCC-2998 112.68
HCT-116 76.14
HCT-15 65.62
HT29 52.57
KM12 96.01
SW-620 80.32
CNS Cancer
SF-268 93.89
SF-295 114.24
SF-539 92.56
SNB-19 104.34
SNB-75 96.41
U251 83.80
Melanoma
LOX IMVI 101.14
MALME-3M 143.93
M14 122.67
MDA-MB-435 101.06
SK-MEL-2 111.13
SK-MEL-28 115.10
SK-MEL-5 92.09
UACC-257 106.87
UACC-62 100.63
Ovarian Cancer
IGROV1 88.13
OVCAR-3 100.36
OVCAR-4 107.16
OVCAR-5 139.15
OVCAR-8 97.35
NCI/ADR-RES 97.38
SK-OV-3 126.60
Renal Cancer
786-0 55.81
A498 61.73
ACHN 95.17
CAKI-1 71.89
RXF 393 49.36
SN12C 102.96
TK-10 129.14
UO-31 57.69
Prostate Cancer
PC-3 89.42
DU-145 103.51
Breast Cancer
MCF7 90.32
MDA-MB-231/ATCC 92.06
HS 578T 96.87
BT-549 109.09
T-47D 99.48
MDA-MB-468 90.05
Mean 93.53
Delta 57.98
Range 108.38
150
100
50
0
-50
-100
-150

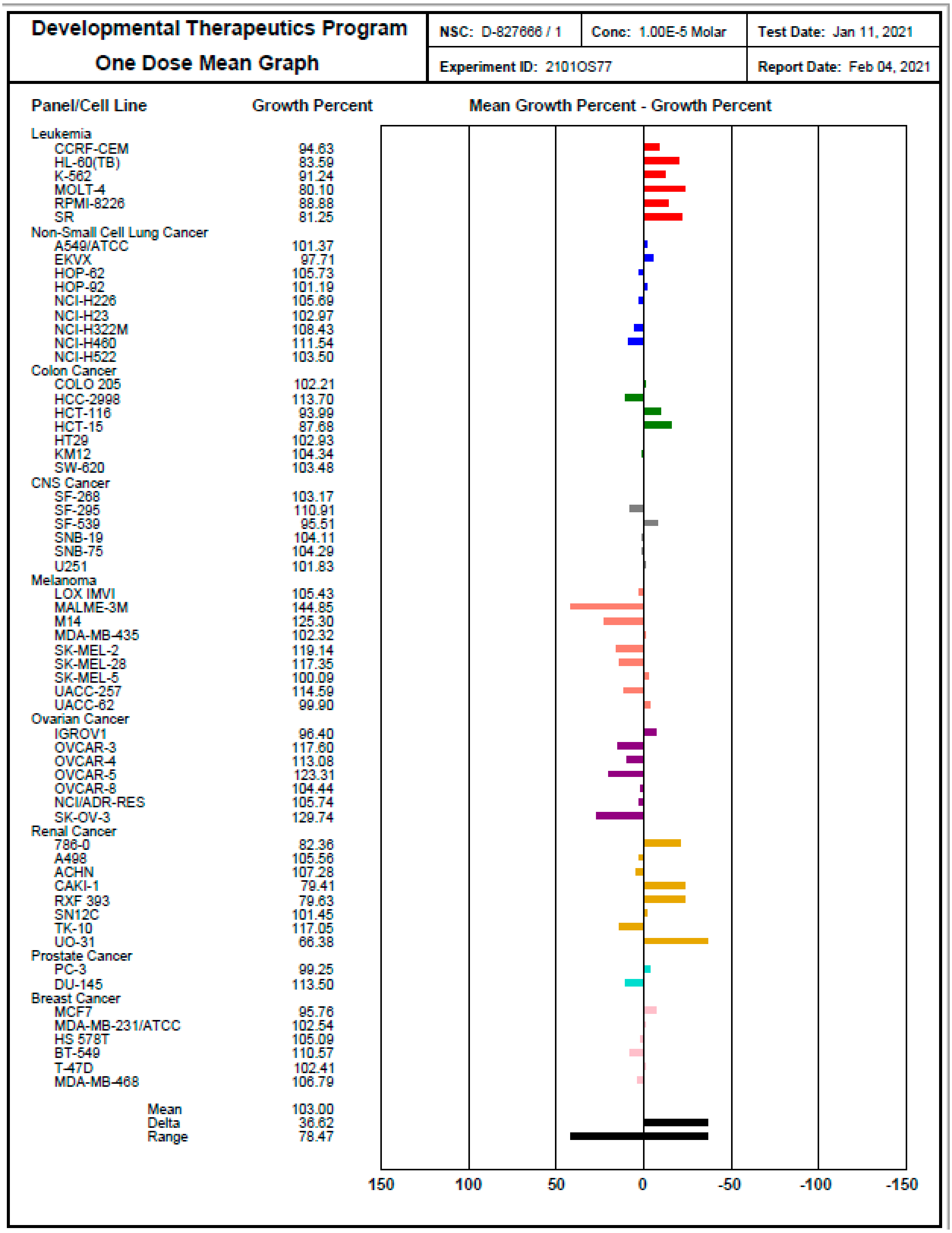
Developmental Therapeutics Program
One Dose Mean Graph
NSC: D-827666 / 1
Conc: 1.00E-5 Molar
Test Date: Jan 11, 2021
Experiment ID: 2101OS77
Report Date: Feb 04, 2021
Panel/Cell Line
Growth Percent
Mean Growth Percent - Growth Percent
Leukemia
CCRF-CEM 94.63
HL-60(TB) 83.59
K-562 91.24
MOLT-4 80.10
RPMI-8226 88.88
SR 81.25
Non-Small Cell Lung Cancer
A549/ATCC 101.37
EKVX 97.71
HOP-62 105.73
HOP-92 101.19
NCI-H226 105.69
NCI-H23 102.97
NCI-H322M 108.43
NCI-H460 111.54
NCI-H522 103.50
Colon Cancer
COLO 205 102.21
HCC-2998 113.70
HCT-116 93.99
HCT-15 87.68
HT29 102.93
KM12 104.34
SW-620 103.48
CNS Cancer
SF-268 103.17
SF-295 110.91
SF-539 95.51
SNB-19 104.11
SNB-75 104.29
U251 101.83
Melanoma
LOX IMVI 105.43
MALME-3M 144.85
M14 125.30
MDA-MB-435 102.32
SK-MEL-2 119.14
SK-MEL-28 117.35
SK-MEL-5 100.09
UACC-257 114.59
UACC-62 99.90
Ovarian Cancer
IGROV1 96.40
OVCAR-3 117.60
OVCAR-4 113.08
OVCAR-5 123.31
OVCAR-8 104.44
NCI/ADR-RES 105.74
SK-OV-3 129.74
Renal Cancer
786-0 82.36
A498 105.56
ACHN 107.28
CAKI-1 79.41
RXF 393 79.63
SN12C 101.45
TK-10 117.05
UO-31 66.38
Prostate Cancer
PC-3 99.25
DU-145 113.50
Breast Cancer
MCF7 95.76
MDA-MB-231/ATCC 102.54
HS 578T 105.09
BT-549 110.57
T-47D 102.41
MDA-MB-468 106.79
Mean 103.00
Delta 36.62
Range 78.47
150
100
50
0
-50
-100
-150

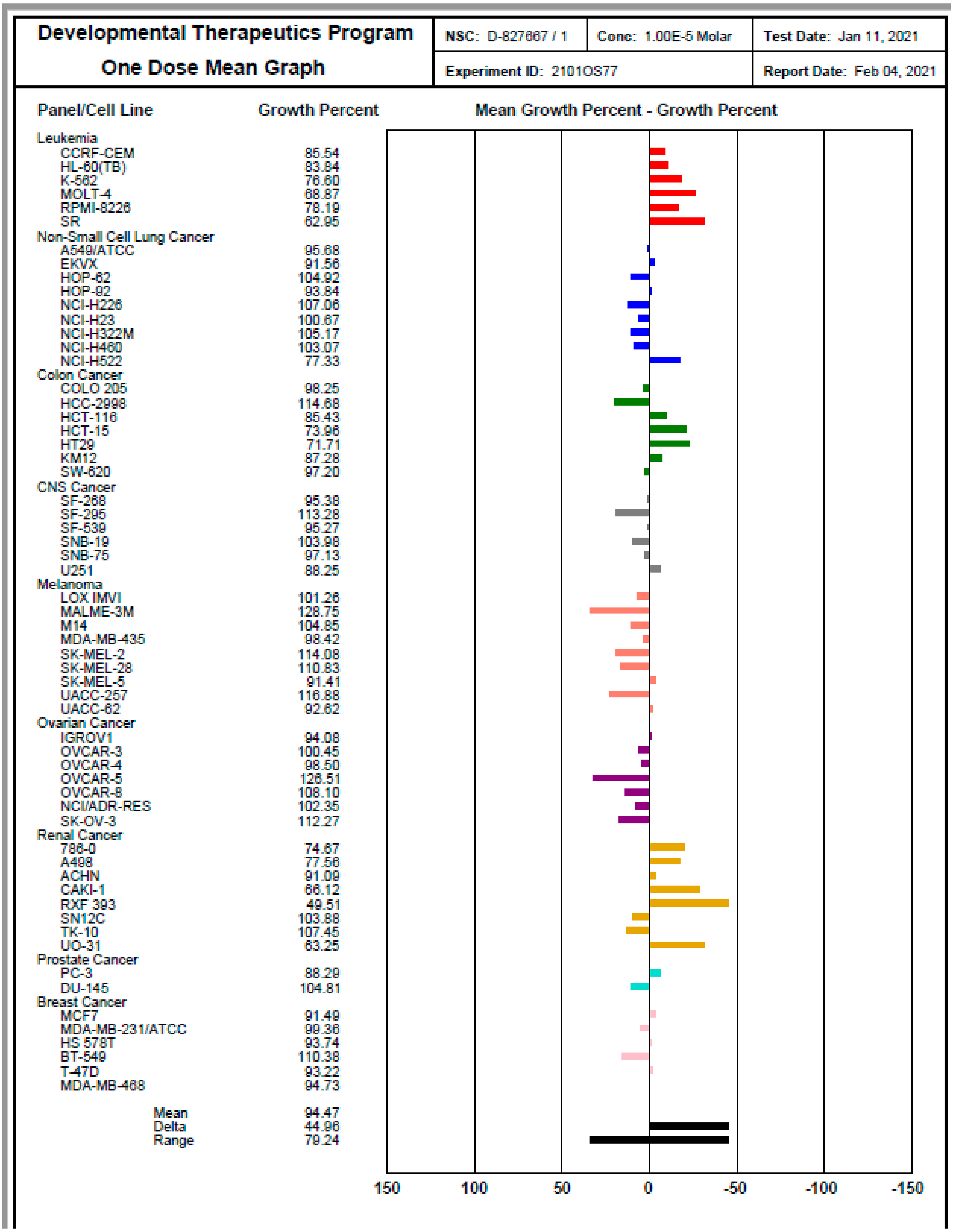
Developmental Therapeutics Program
One Dose Mean Graph
NSC: D-827667 / 1
Conc: 1.00E-5 Molar
Test Date: Jan 11, 2021
Experiment ID: 2101OS77
Report Date: Feb 04, 2021
Panel/Cell Line
Growth Percent
Mean Growth Percent - Growth Percent
Leukemia
CCRF-CEM 85.54
HL-60(TB) 83.84
K-562 76.60
MOLT-4 68.87
RPMI-8226 78.19
SR 62.95
Non-Small Cell Lung Cancer
A549/ATCC 95.68
EKVX 91.56
HOP-62 104.92
HOP-92 93.84
NCI-H226 107.06
NCI-H23 100.67
NCI-H322M 105.17
NCI-H460 103.07
NCI-H522 77.33
Colon Cancer
COLO 205 98.25
HCC-2998 114.68
HCT-116 85.43
HCT-15 73.96
HT29 71.71
KM12 87.28
SW-620 97.20
CNS Cancer
SF-268 95.38
SF-295 113.28
SF-539 95.27
SNB-19 103.98
SNB-75 97.13
U251 88.25
Melanoma
LOX IMVI 101.26
MALME-3M 128.75
M14 104.85
MDA-MB-435 98.42
SK-MEL-2 114.08
SK-MEL-28 110.83
SK-MEL-5 91.41
UACC-257 116.88
UACC-62 92.62
Ovarian Cancer
IGROV1 94.08
OVCAR-3 100.45
OVCAR-4 98.50
OVCAR-5 126.51
OVCAR-8 108.10
NCI/ADR-RES 102.35
SK-OV-3 112.27
Renal Cancer
786-0 74.67
A498 77.56
ACHN 91.09
CAKI-1 66.12
RXF 393 49.51
SN12C 103.88
TK-10 107.45
UO-31 63.25
Prostate Cancer
PC-3 88.29
DU-145 104.81
Breast Cancer
MCF7 91.49
MDA-MB-231/ATCC 99.36
HS 578T 93.74
BT-549 110.38
T-47D 93.22
MDA-MB-468 94.73
Mean 94.47
Delta 44.96
Range 79.24
150
100
50
0
-50
-100
-150

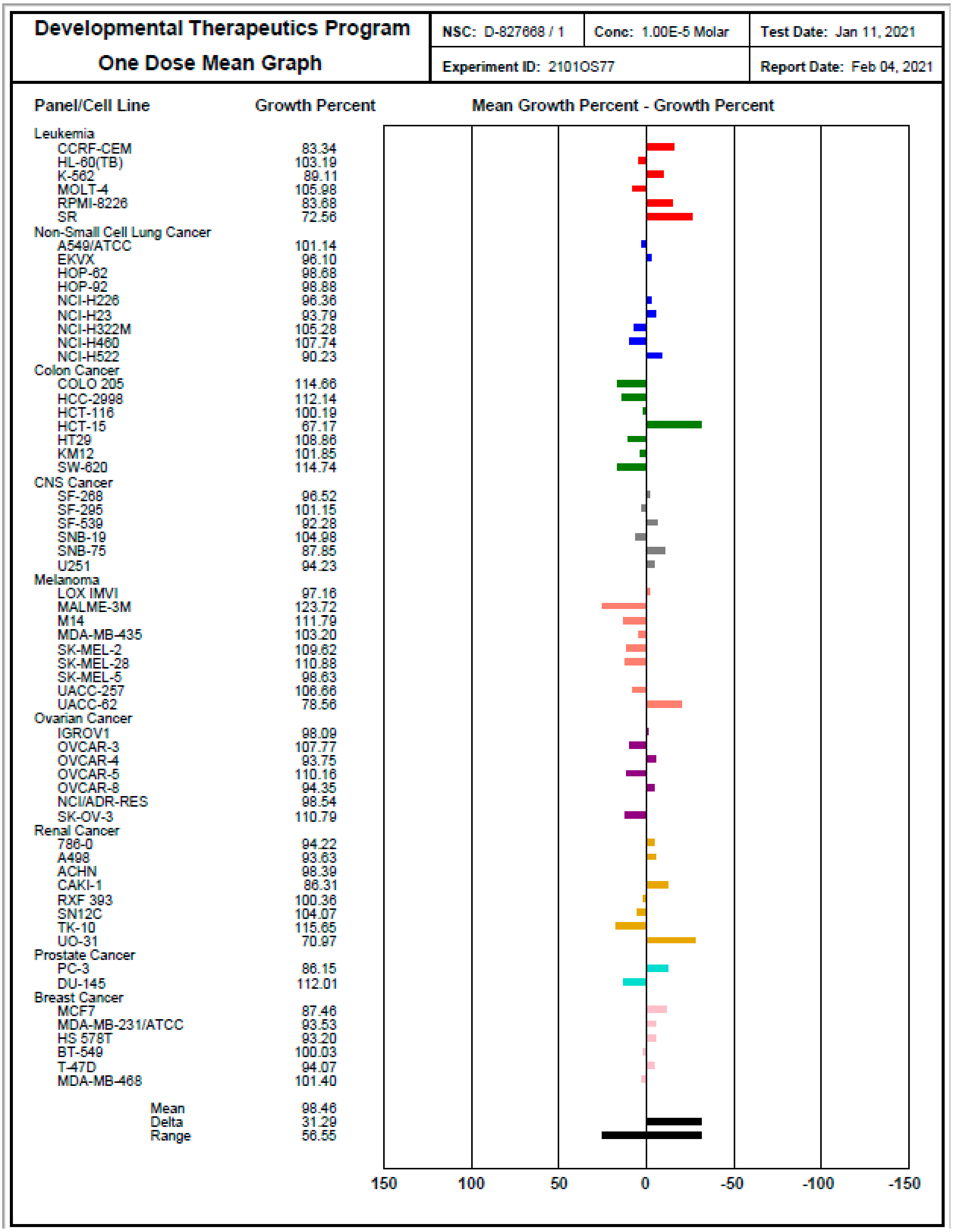
Developmental Therapeutics Program
One Dose Mean Graph
NSC: D-827668 / 1
Conc: 1.00E-5 Molar
Test Date: Jan 11, 2021
Experiment ID: 2101OS77
Report Date: Feb 04, 2021
Panel/Cell Line
Growth Percent
Mean Growth Percent - Growth Percent
Leukemia
CCRF-CEM 83.34
HL-60(TB) 103.19
K-562 89.11
MOLT-4 105.98
RPMI-8226 83.68
SR 72.56
Non-Small Cell Lung Cancer
A549/ATCC 101.14
EKVX 96.10
HOP-62 98.68
HOP-92 98.88
NCI-H226 96.36
NCI-H23 93.79
NCI-H322M 105.28
NCI-H460 107.74
NCI-H522 90.23
Colon Cancer
COLO 205 114.66
HCC-2998 112.14
HCT-116 100.19
HCT-15 67.17
HT29 108.86
KM12 101.85
SW-620 114.74
CNS Cancer
SF-268 96.52
SF-295 101.15
SF-539 92.28
SNB-19 104.98
SNB-75 87.85
U251 94.23
Melanoma
LOX IMVI 97.16
MALME-3M 123.72
M14 111.79
MDA-MB-435 103.20
SK-MEL-2 109.62
SK-MEL-28 110.88
SK-MEL-5 98.63
UACC-257 106.66
UACC-62 78.56
Ovarian Cancer
IGROV1 98.09
OVCAR-3 107.77
OVCAR-4 93.75
OVCAR-5 110.16
OVCAR-8 94.35
NCI/ADR-RES 98.54
SK-OV-3 110.79
Renal Cancer
786-0 94.22
A498 93.63
ACHN 98.39
CAKI-1 86.31
RXF 393 100.36
SN12C 104.07
TK-10 115.65
UO-31 70.97
Prostate Cancer
PC-3 86.15
DU-145 112.01
Breast Cancer
MCF7 87.46
MDA-MB-231/ATCC 93.53
HS 578T 93.20
BT-549 100.03
T-47D 94.07
MDA-MB-468 101.40
Mean 98.46
Delta 31.29
Range 56.55
150
100
50
0
-50
-100
-150

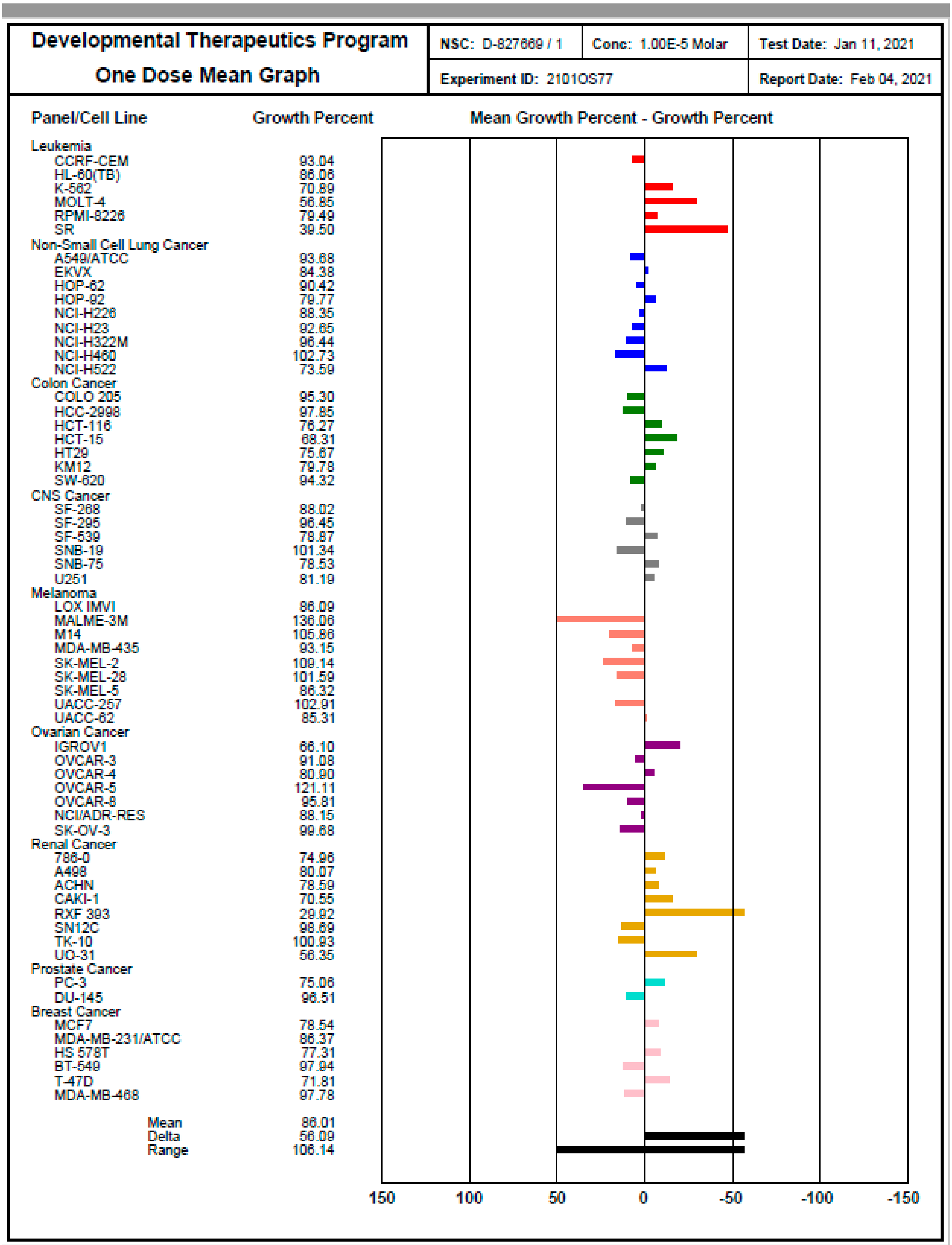
Developmental Therapeutics Program
One Dose Mean Graph
NSC: D-827669 / 1
Conc: 1.00E-5 Molar
Test Date: Jan 11, 2021
Experiment ID: 2101OS77
Report Date: Feb 04, 2021
Panel/Cell Line
Growth Percent
Mean Growth Percent - Growth Percent
Leukemia
CCRF-CEM 93.04
HL-60(TB) 86.06
K-562 70.89
MOLT-4 56.85
RPMI-8226 79.49
SR 39.50
Non-Small Cell Lung Cancer
A549/ATCC 93.68
EKVX 84.38
HOP-62 90.42
HOP-92 79.77
NCI-H226 88.35
NCI-H23 92.65
NCI-H322M 96.44
NCI-H460 102.73
NCI-H522 73.59
Colon Cancer
COLO 205 95.30
HCC-2998 97.85
HCT-116 76.27
HCT-15 68.31
HT29 75.67
KM12 79.78
SW-620 94.32
CNS Cancer
SF-268 88.02
SF-295 96.45
SF-539 78.87
SNB-19 101.34
SNB-75 78.53
U251 81.19
Melanoma
LOX IMVI 86.09
MALME-3M 136.06
M14 105.86
MDA-MB-435 93.15
SK-MEL-2 109.14
SK-MEL-28 101.59
SK-MEL-5 86.32
UACC-257 102.91
UACC-62 85.31
Ovarian Cancer
IGROV1 66.10
OVCAR-3 91.08
OVCAR-4 80.90
OVCAR-5 121.11
OVCAR-8 95.81
NCI/ADR-RES 88.15
SK-OV-3 99.68
Renal Cancer
786-0 74.96
A498 80.07
ACHN 78.59
CAKI-1 70.55
RXF 393 29.92
SN12C 98.69
TK-10 100.93
UO-31 56.35
Prostate Cancer
PC-3 75.06
DU-145 96.51
Breast Cancer
MCF7 78.54
MDA-MB-231/ATCC 86.37
HS 578T 77.31
BT-549 97.94
T-47D 71.81
MDA-MB-468 97.78
Mean 86.01
Delta 56.09
Range 106.14
150
100
50
0
-50
-100
-150

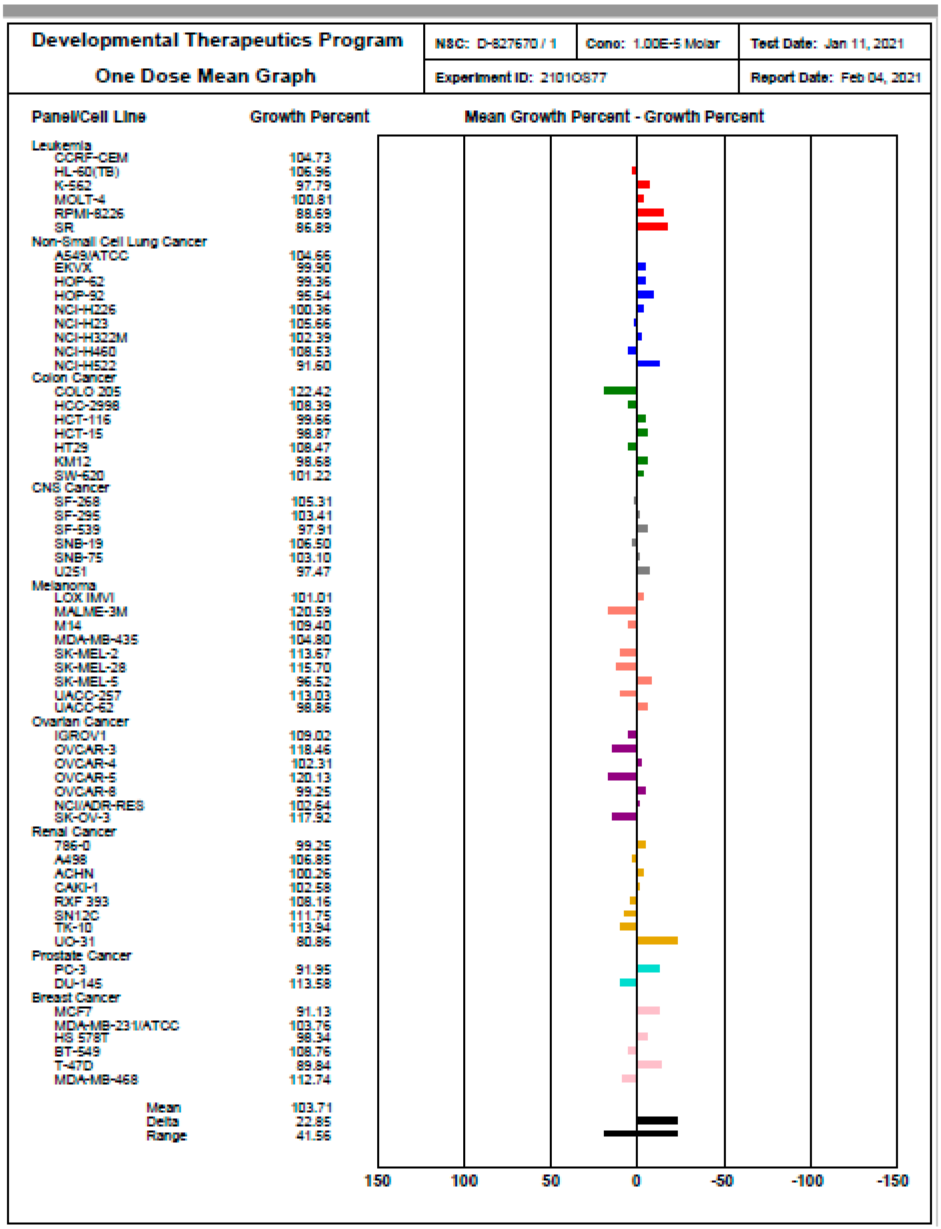

8. ADME (SwissADME)

Calculations were performed by the SwissADME web service http://www.swissadme.ch developed by the Molecular Modeling Group of the Swiss Institute of Bioinformatics that gives free access to a pool of fast yet robust predictive models for small molecules pharmacokinetic properties. (Sci. Rep. 2017, 7, 42717).
